# ChIP happens: from biochemical origins to the modern omics toolbox for understanding steroid hormone receptors

**DOI:** 10.1042/BCJ20253216

**Published:** 2026-02-02

**Authors:** Thomas F. Grimes, Jacob Pope, Jack Stenning, Taylor E. Smith, David G. Kent, Simon Baker, William J. Brackenbury, Lianne I. Willems, Andrew N. Holding

**Affiliations:** 1 York Biomedical Research Institute, University of York, York, North Yorkshire, YO10 5DD, United Kingdom; 2 Department of Biology, University of York, York, North Yorkshire, YO10 5DD, United Kingdom; 3 Centre for Blood Research, University of York, York, North Yorkshire, YO10 5DD, United Kingdom; 4 Jack Birch Cancer Research Unit, University of York, York, North Yorkshire, YO19 5DD, United Kingdom; 5 York Structural Biology Laboratory, Department of Chemistry, University of York, York, North Yorkshire, YO10 5DD, United Kingdom

**Keywords:** androgens, biochemical techniques and resources, chromatin, cortisol, DNA sequencing, epigenomics, estrogens, genomics, mass spectrometry, steroids

## Abstract

Nuclear steroid hormone receptors (SHRs) are ligand-activated transcription factors that mediate cellular responses to steroid hormones (SHs) through regulating gene expression. Understanding the SHR function is crucial for elucidating SH-driven physiology and pathology, including their roles in normal development, metabolism and reproduction, alongside their aberrant function in cancer, endocrine disorders and inflammatory diseases. Investigating the mechanisms that underscore SHR signalling and regulation is therefore essential for advancing our knowledge of both normal physiology and disease and is vital to the development of novel therapeutic strategies. In this review, we examine a range of methods for studying SHR interactions with chromatin and coregulator proteins, from classical biochemical assays to more advanced approaches such as PL-MS, RIME and ChIP. We also highlight potential future innovations in the field, including *in situ* Calling Cards and UV-induced photocross-linking RIME (UVXL-RIME), that may overcome current methodological limitations, in turn enabling the study of SHRs in increasingly physiologically relevant contexts.

## Structure and function of steroid hormones and receptors

### Steroid hormones

Steroid hormones (SHs) are a group of lipophilic molecules, naturally produced by all eukaryotes. In vertebrates, SHs are divided into five groups based on their function, structure and biosynthetic route from cholesterol: mineralocorticoids, glucocorticoids (GCs), estrogens, androgens and progestins (also known as progestogens) [1]. Of these, mineralocorticoids and GCs are collectively known as corticosteroids, while androgens, estrogens and progestins are termed the sex hormones.

**Figure 1:**
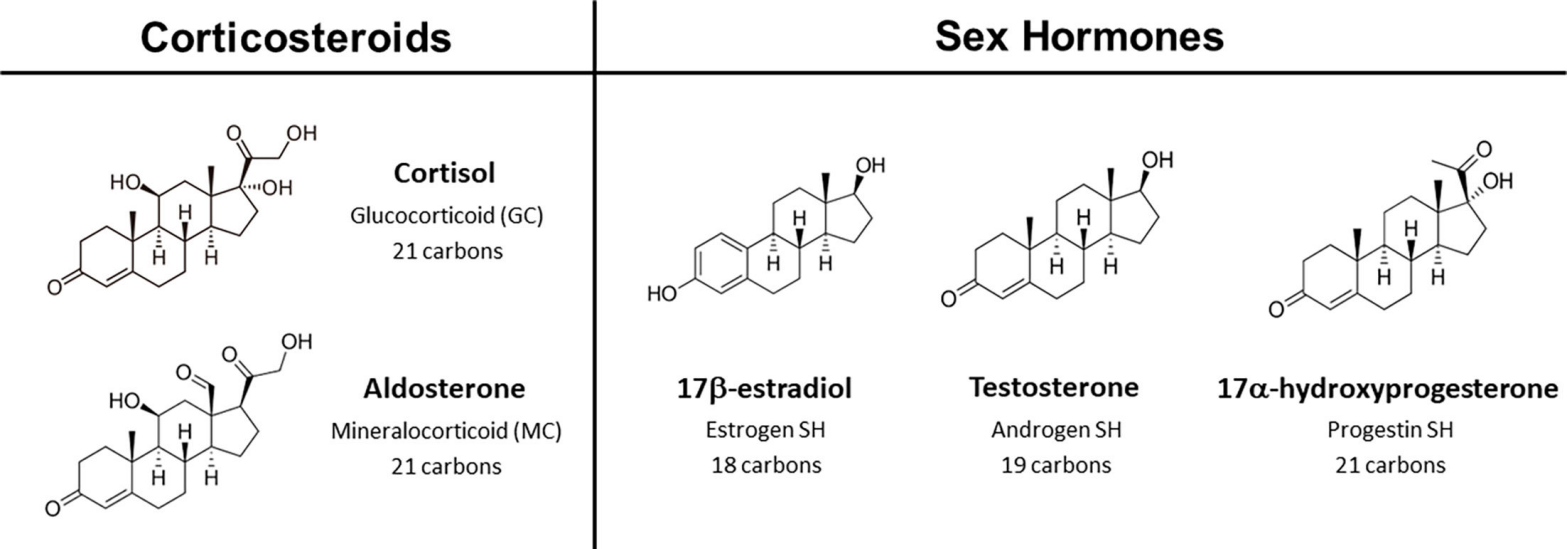
Representative 2D chemical structures of the five steroid hormone (SH) classes. Corticosteroids, including glucocorticoids (e.g. cortisol) and mineralocorticoids (e.g. aldosterone) possess a tetracyclic ring system comprising 21 carbon atoms. In contrast, estrogens (e.g. 17β-estradiol), androgens (e.g. testosterone) and progestins (e.g. 17a-hydroxyprogesterone) contain tetracyclic structures composed of 18, 19 and 21 carbon atoms, respectively.

All corticosteroids possess a polycyclic system composed of 21 carbon atoms (Figure 1), although their biological activities vary markedly [1–8]. GCs, such as cortisone, play key roles in modulating the immune system towards an anti-inflammatory state [2], regulating protein, sugar and fat metabolism [1,3–5], and controlling cell growth, differentiation and apoptosis [6,7]. In contrast, mineralocorticoids (MCs) like aldosterone are involved in fluid/electrolyte balance and blood pressure regulation [1,8]. Interestingly, certain corticosteroids can act as both GCs and MCs; for instance, cortisol predominantly functions as a GC but displays MC activity in low aldosterone environments [1,9].

Androgens, estrogens and progestins differ both in the number of carbon atoms within their polycyclic systems (Figure 1) and in their biological activities [1,10–18]. The primary function of sex hormones is to promote normal female (estrogens and progestins) or male (androgens) development and reproductive function [1]. These effects depend on the ratio of female to male sex hormones, which are present at varying levels in both sexes. Beyond reproduction, sex hormones are also implicated in bone and muscle development [10,11]; cardiovascular development [12–14]; metabolism [15,16]; and thyroid function [17,18].

In humans, corticosteroids are predominantly synthesised in the adrenal glands, while sex hormones are mainly produced in the gonads [1,19]. After synthesis, SHs are secreted into the bloodstream, where they bind to carrier proteins [20,21] that facilitate transport to distant tissues. SHs subsequently dissociate from these carriers [22], and either bind directly to membrane-associated steroid hormone receptors (SHRs) [23–25], or diffuse across cell-surface membranes to activate intracellular SHRs [26]. Consequently, SHs primarily act through long-range endocrine signalling mechanisms, although extra-adrenal and extra-gonadal SH biosynthesis does occur [27,28] enabling short-range signalling.

## An overview of SHRs

### Historical milestones in SHR research

SHRs were first characterised between 1960 and 1990 through a series of consecutive experiments, as reviewed by Beato et al. [26]. The first of these studies, published by Jensen and Jacobsen [29,30], used radiolabeled 17-estradiol to identify estrogen-responsive cells in rats, including those in the mammary glands and uterus. Simultaneous work by Clever and Karlson [31] showed that ecdysone, an insect SH, induced giant chromosomal puff formation (unwound DNA sections indicative of active transcription). Collectively, this seminal work implied the presence of intracellular SHRs in specific cells that regulate gene transcription following SH stimulation.

Subsequently, Toft and Gorski [32] isolated the rat estrogen receptor (rER) from the soluble fraction of the uterus, showing rER binds specifically to estrogen SHs, but not to androgens, progestins, or corticosteroids. ERs were later isolated from the uteri of other species, including mice (mER) [33] and chickens (cER) [34], with these studies reporting a molecular weight of ~65 kDa, and a low nanomolar affinity (*K_d_* ~1 × 10^-10^ M) for estrogenic SHs.

Concurrent work by Noteboom, Gorski [35] and Jensen [36] led to the proposition of a two-step model for SHR activation. According to this framework, SHs diffuse across the cell-surface membrane and bind, with a high affinity, to their cognate SHR in the cytoplasm. This binding activates the SHR, triggering nuclear translocation, and in turn, the expression of specific hormone-responsive genes.

Major advances in the field of SHRs next emerged in the 1980s, when the human glucocorticoid receptor (hGR) [37,38], human androgen receptor (hAR) [39,40], hER [41–43], and various SH-responsive genes [44,45] had been successfully cloned from cDNA libraries. These pivotal studies led to the identification of the first hormonal response elements (HREs) – palindromic DNA sequences upstream of many SH-responsive genes that serve as SHR binding sites [46–48] and are now accepted to function within extended promoter-enhancer regulatory networks [49–51].

### Phylogeny and structure of SHRs

Phylogenetically, SHRs belong to the nuclear receptor (NR) superfamily of proteins. This review article will focus on the NR3 subfamily of nuclear receptors, which traditionally were characterised as class I homodimers [52]. However, recent work has shown that NR3 SHRs can adopt a diverse range of quaternary structures, including heterodimers and higher-order assemblies [53]. In vertebrates, the NR3 subfamily comprises six SHRs encoded by distinct genes [26] – the glucocorticoid receptor (GR), progesterone receptor (PR), estrogen receptors (ERα and ERβ), androgen receptor (AR) and mineralocorticoid receptor (MR) – with additional lineage-specific paralogues present in certain teleost (bony fish) species [54].

**Figure 2:**
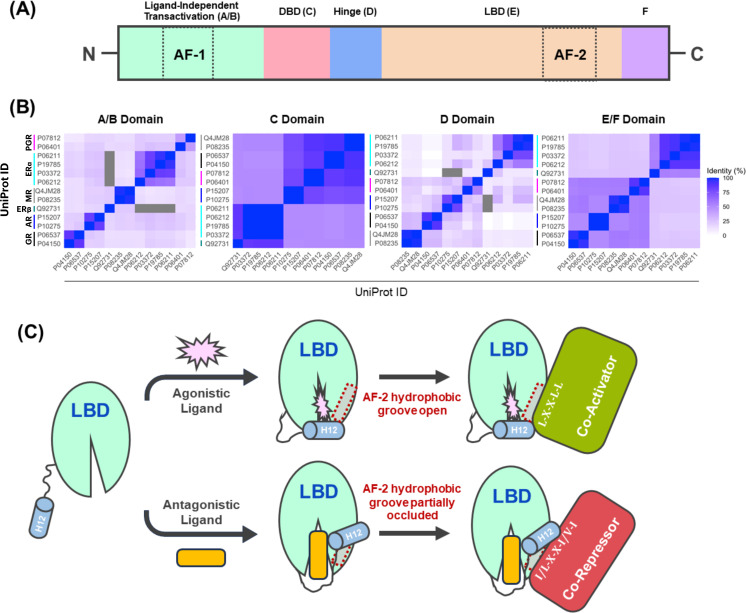
Steroid hormone receptor (SHR) domains. **(A**) Schematic of generic SHR protein domain structure. Each SHR possesses an *N*-terminal transactivation domain (A/B domain) that contains the ligand-independent activation function (AF-1), a DNA-binding domain (DBD; C domain), a hinge domain (D domain), a ligand-binding domain (LBD; E domain) that encompasses activation function 2 (AF-2), and a *C*-terminal F domain. (**B**) Heatmaps showing SHR per-domain sequence conservation. SHR amino acid sequences (UniProt IDs shown) were retrieved, domain boundaries annotated using InterPro, and sequences aligned using ClustalOMEGA. Identity matrices were subsequently plotted as heatmaps in R. The E and F domains were combined as not all SHRs contain distinct F domains.

Although each SHR has unique tissue expression patterns and biological functions [55,56], they generally share a common modular structure comprising five domains (Figure 2A): (i) an *N*-terminal transactivation domain encompassing the ligand-independent transcriptional activation function (AF-1) [57,58]; (ii) a DNA binding domain (DBD); (iii) a hinge domain containing the nuclear localisation signal; (iv) a ligand binding domain (LBD) that harbours the ligand-dependent transcriptional activation function (AF-2) [57,58]; and (v) a *C*-terminal domain that helps distinguish agonistic from antagonistic SHs and mediates protein-protein interactions. Classically, these domains have been labelled A/B, C, D, E and F respectively (Figure 2A) [26,59,60]. Notably, the C and E domains are well conserved between SHRs, while the A/B, D and F domains show greater variability in their length and amino acid sequence (Figure 2B) [59,60].

**Figure 3:**
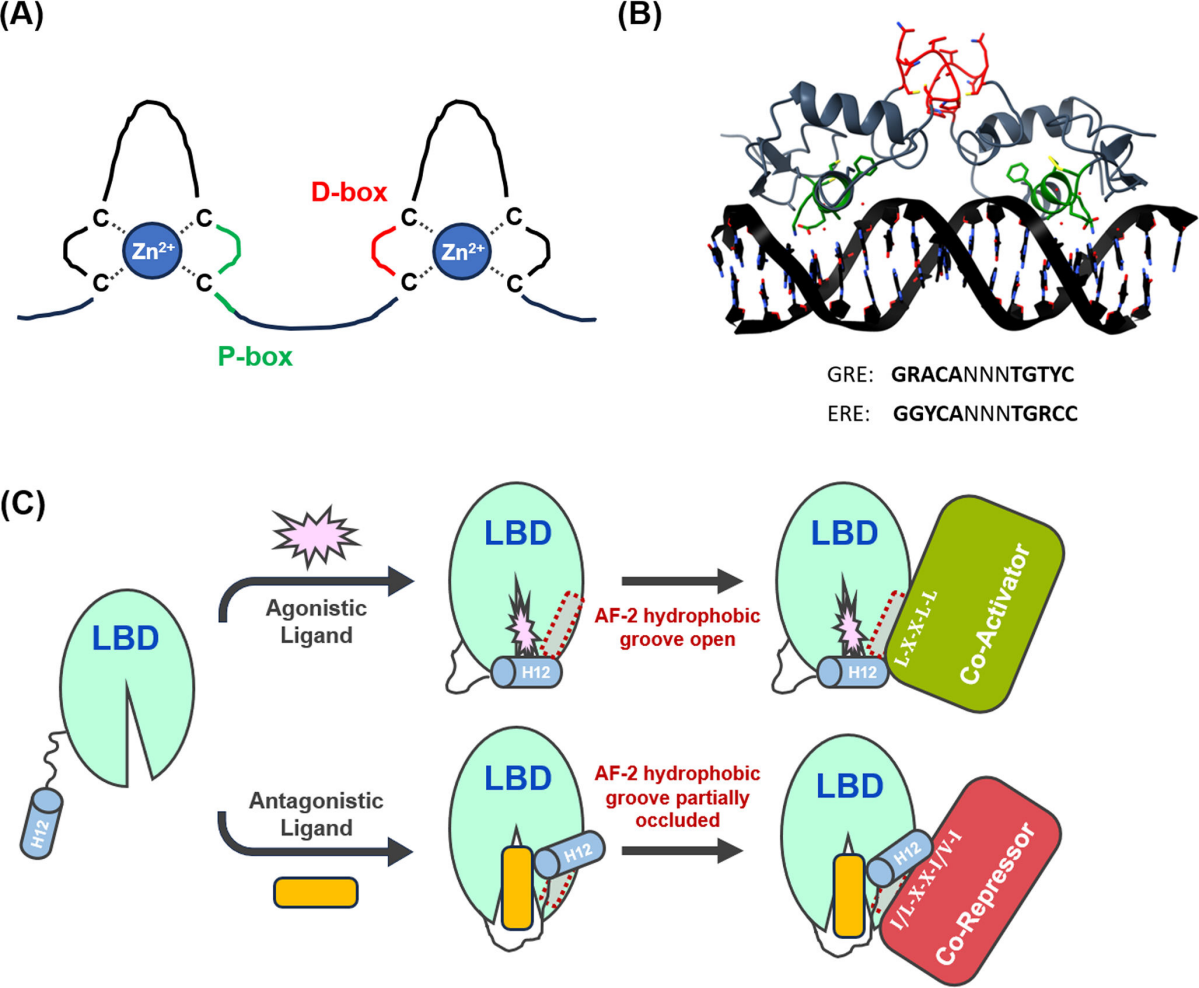
Structural and functional overview of steroid hormone receptor DNA-binding (DBD) and ligand-binding (LBD) domains. **(A**) Illustration of the two SHR DBD Zinc-finger motifs (ZFMs). Each ZFM comprises four cysteines that co-ordinate a single Zn^2+^ ion in a tetrahedral arrangement. The amino acids that form the P-box, responsible for DNA binding, are coloured in green, while those forming the D-box, which facilitate SHR dimerisation, are shown in red. (**B**) Crystal structure of homodimeric hERα DBDs in complex with an estrogen response element (ERE), PDB ID: 1HCQ [62]. The P-box amino acid residues are coloured in green, with the D-box residues depicted in red. The consensus sequences for the canonical glucocorticoid response element (GRE) and canonical ERE are shown, with the separate half-sites highlighted in bold. (**C**) Ligand-induced conformational dynamics of SHR LBDs. Upon agonistic ligand binding (e.g. 17β-estradiol for hERα), LBD alpha-helix H12 encloses the ligand binding cavity, forming the activation function 2 (AF-2) surface. Transcriptional coactivators are subsequently recruited to a hydrophobic groove within AF-2 via nuclear receptor (NR) box motifs with the consensus sequence L-X-X-L-L. In contrast, the binding of antagonistic ligands (e.g. tamoxifen for hERα) triggers H12 to partially occlude the AF-2 hydrophobic groove, blocking coactivator binding. Instead, transcriptional corepressors are recruited to the AF-2 groove via Co-Repressor nuclear receptor (CoRNR) box motifs possessing the consensus sequence I/L-X-X-I/V-I. R, purine base (A/G); Y, pyrimidine base (C/T); N, any nucleotide (A/G/C/T).

The DBD of all SHRs contains two zinc-finger motifs (ZFMs), termed the proximal (P) and distal (D) boxes. Each ZFM contains four cysteine residues that co-ordinate a Zn^2+^ ion in a tetrahedral configuration [61] (Figure 3A). The P-box consists of three amino acid residues that adopt an alpha-helical arrangement, thereby positioning their side chains within the DNA major groove to contact specific bases in hormonal response elements (HREs) [26,61,63]. In contrast, the D-box aids SHR dimerisation [26,61], aligning two P-boxes with their respective HRE half-sites (Figure 3B). Notably, GR, MR, AR and PR all recognise a common HRE known as the glucocorticoid response element (GRE) [46–48,64–66], while ER binds to the distinct estrogen response element (ERE) (Figure 3B) [67,68]. The use of a common motif suggests the genomic and cellular context surrounding GREs helps to determine which SHR is recruited, with the accessibility of the chromatin, the presence of other transcription factors or regulatory elements, cell-type specific expression of SHRs, and SH ligand availability all playing a role [69].

The LBD is highly conserved among SHRs, possessing a well-defined 3D structure of 12 amphipathic α-helices arranged into a large hydrophobic cavity for ligand binding [26,70–72]. While the composition of this cavity varies between SHRs to enable interactions with their cognate SHs, the overall structure and dynamics of the LBD are well preserved [70–72]. Following agonistic ligand binding, α-helix 12 (H12) is repositioned over the entrance of the ligand binding cavity, forming the activation function 2 (AF-2) surface (Figure 3C) [70–72]. Minor contributions to this interaction surface are likely also made by adjacent α-helices [70–73]. Transcriptional coactivators are subsequently recruited to a hydrophobic groove within AF-2 through nuclear receptor (NR) box motifs containing the consensus sequence L-X-X-L-L (Figure 3C) [70–72,74]. Conversely, antagonistic ligands force AF-2 into a repressive structure, where H12 partially occludes the hydrophobic groove [70–72]. This conformational change blocks coactivator recruitment, although transcriptional corepressors can still bind to the AF-2 groove via Co-Repressor Nuclear Receptor (CoRNR) box motifs with the consensus sequence I/L-X-X-I/V-I (Figure 3C) [75]. Regardless of the ligand type, SHRs retain a large hydrophobic dimerisation interface within their LBD, primarily formed by α-helices H8-H12 [70–72,76].

The *N*-terminal ligand-independent transactivation domain (A/B domain) is poorly conserved among SHRs and is characterised by its intrinsic disorder and structural flexibility [77–79]. The domain houses activation function 1 (AF-1), which recruits transcriptional coregulators to modulate target gene expression independently of SH-binding. AF-1 can adopt several active conformations that permit coregulator recruitment, influenced by adjacent transcription factors [77–79], and certain post-translational modifications, such as phosphorylation [80–87]. Although AF-1 and AF-2 can operate independently, full transcriptional control requires both regions to work synergistically [88,89]. Beyond contributing to transcriptional regulation, the intrinsically disordered regions (IDRs) of the A/B domain have recently been implicated in chromatin binding and searching [90,91].

The hinge (D) domain and F-domain, both poorly conserved between SHRs (Figure 2B), play key roles in receptor function. The hinge domain contains the nuclear localisation signal (NLS) peptide, which enables the nuclear import of SHRs [92]. The D-domain also acts as a flexible linker between AF-1 and AF-2, supporting their synergy and allowing complete SHR transcriptional control [89,92]. In contrast, the function of the F-domain is less well understood, although it seems to help distinguish between agonistic and antagonistic ligands [93–95], and may support transcriptional coregulator recruitment by AF-1 and AF-2 [93–95].

### Isoforms of SHRs

Alongside variation in the A/B, D and F domains of SHRs, their diversity is further expanded by distinct protein isoforms. This was first demonstrated by Kastner et al*.* [96] who identified two variants of human progesterone receptor (hPR), PR-A and PR-B. These isoforms are generated by alternative promoter usage during *PGR* transcription [96], yielding a full-length variant (hPR-B), and a truncated form (hPR-A) lacking 164 residues at the *N*-terminal. Both isoforms retain high progestin-binding affinities, can regulate PR target gene expression [96] and are present at approximately equal levels in normal human luminal mammary epithelial cells [97]. However, PR-A and PR-B differ in their abilities to recruit coregulators [98–101], with PR-B possessing a third activation function (AF-3) that can function either autonomously or synergistically with AF-1 and AF-2 [100]. In addition, in malignant breast lesions, PR-A is often overexpressed, skewing the PR-A:PR-B ratio and, in turn, implicating PR-A in luminal breast cancer metastasis and invasion [97,102].

Like hPR, hAR, hER and hGR also exist as two predominant variants [26]. For hAR, the B isoform is full-length, whereas hAR-A lacks 188 residues at the *N*-terminus due to an internal translation initiation site in the *AR* mRNA transcript [103,104]. Although hAR-A is expressed at significantly lower levels than hAR-B in most cells [103,104], including normal and malignant prostate tissue [105], both isoforms bind androgens [26,103,104] and regulate AR target gene expression [106]. However, the truncated A/B domain of hAR-A markedly impairs its ability to recruit coregulators, thereby diminishing transcriptional control by the A isoform. This effect has been shown in osteoblasts and fibroblasts, where AR-A was less effective than AR-B at inducing AR target gene expression and promoting cellular proliferation [107].

Unlike other SHRs, hERα and hERβ are not splice variants of a single gene, but rather homologous receptors encoded by two separate genes (*ESR1* and *ESR2* respectively) [108–110]. Both receptors give rise to multiple truncated isoforms via alternative splicing, differential promoter usage and internal translation initiation [111–115], although truncated ER variants generally exhibit reduced transactivation capacity when compared with full-length ERs [111–115]. Nonetheless, certain truncated ERα isoforms (e.g. cds11, cds16) can still regulate target gene expression, with cds11 showing enhanced nuclear localisation relative to full-length ERα [111]. Moreover, the hERα cds1 variant, which lacks the complete LBD, has shown resistance to fulvestrant treatment, although it does not display transcriptional activity [111]. Turning to the full-length receptors, hERα and hERβ diverge in several biologically meaningful ways despite sharing structural homology and regulating target gene expression through similar mechanisms. For instance, hERα/hERβ exhibit unique cellular expression patterns [56]; hERα is only present in luminal mammary epithelial cells, while hERβ is expressed in epithelial and myoepithelial cells. The full-length ERs also have different affinities for non-consensus EREs [68,116], with both overlapping and unique genomic binding sites being reported [117]. Additionally, ERα drives more potent transactivation than ERβ [89,118,119], likely due to the impaired function of the hERβ AF-1 region and hinge domain [89]. Collectively, these observations reinforce the proposal that hERβ functions as a ligand-activated tumour suppressor [56,120], while hERα is generally accepted to be a ligand-dependent oncogene [121]. However, efforts to define the biological role of hERβ have been hindered due to issues with the availability of reliable, well-validated experimental reagents [122].

The two main hGR isoforms, GRα and GRβ, are produced from a single gene via alternative splicing [26,37,123], although truncated variants that regulate unique subsets of genes also arise through various translational mechanisms [124]. Notably, hGRα and hGRβ are identical up to residue 727, after which their sequences diverge, with hGRα being the longer variant (777 *vs* 742 amino acids) [26,37]. GRα is highly conserved as the functional GR isoform across most species [125], while GRβ is generally considered transcriptionally inactive due to a truncated LBD and instead exerts a dominant negative effect on GRα-mediated gene repression [26,126]. Interestingly, the mechanism of GR dimerisation also differs from other SHRs, with early studies reporting this to be reliant on an intermolecular β-sheet motif and a small hydrophobic interface within the LBD [72]. However, more recent work has shown that GR can adopt up to 20 different homodimeric structures depending on the bound ligand and associated transcriptional coregulators [127]. This results in a markedly weaker dimerisation affinity: *Kd* ~2 µM for GR [72,127], compared with *Kd* ~3 nM for ER [128,129].

hMR exists as a single protein isoform encoded by two mRNA variants, hMRα and hMRβ, that arise from alternative splicing and differential promoter usage [130–135]. These variants share identical protein-coding sequences but differ in their 5’ untranslated regions (5’-UTRs) [130–134]. As such, hMRα and hMRβ are thought to have unique stabilities and translational efficiencies *in vivo*, likely influencing the overall hMR protein level within cells [133,136–138]. Less common hMR protein isoforms that originate from alternative splicing have also been reported [130,139–141], although these account for less than 10% of total hMR protein levels [130–135].

## Genomic signalling mechanisms of SHRs

### SHR activation and chromatin association

SHRs can regulate target gene transcription in a direct genomic fashion. In the absence of their cognate ligands, SHRs are sequestered in a high-affinity, transcriptionally inactive state by chaperone (e.g. Hsp90, Hsp70) and co-chaperone (e.g. FKBP51, FKBP52) proteins [142–152]. Depending on the SHR, this inactive chaperone heterocomplex might be found in the nucleus (ER, PR-A) due to constitutively active NLS peptides [151–155], the cytoplasm (GR, AR, MR) [152], or between the two compartments (PR-B) [155]. Regardless of their subcellular localisation, all SHRs undergo near constant nucleocytoplasmic shuttling [151,154–156], probably accounting for the low level of cytoplasmic ERα observed by quantitative immunofluorescence in patient-derived breast tissue [153], and by immunocytochemistry in ERα-expressing uterine (SHM) and mammary (MCF-7) immortalised cell lines [156].

**Figure 4:**
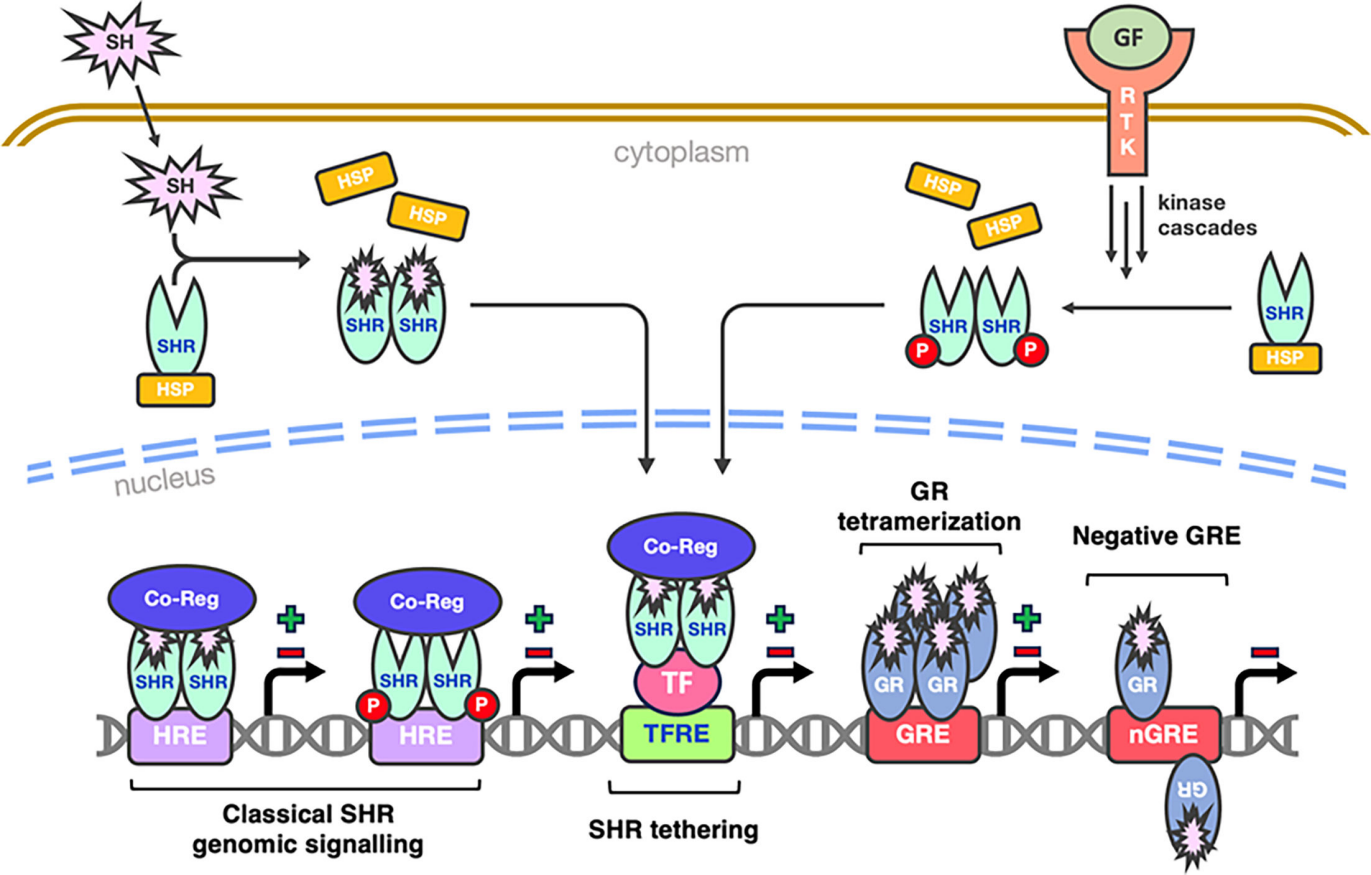
Schematic of genomic steroid hormone receptor (SHR) signalling mechanisms. Inactive SHRs are typically sequestered in the cytoplasm by heat shock proteins (HSPs). SHR activation occurs either via steroid hormone (SH) ligand binding, or through ligand-independent mechanisms such as growth factor (GF)-mediated phosphorylation. Following activation, SHRs dimerise and are translocated into the nucleus, where they recruit transcriptional coregulators to activate (+) or repress (-) target gene expression. Notably, some SHRs (e.g. ER, PR-A) localise to the nucleus in their inactive state due to constitutively active NLS peptides, so do not undergo nuclear translocation (not shown). RTK, receptor tyrosine kinase; Co-Reg, transcriptional coregulator; HRE, hormone response element; TFRE, transcription factor response element; GR, glucocorticoid receptor; GRE, glucocorticoid response element.

Following SH ligand binding (e.g. 17β-estradiol for ERα) [32], SHR monomers experience a major conformational change where H12 of the LBD is repositioned over the entrance of the ligand binding cavity to form the AF-2 interaction surface [70,71,142,143]. This structural shift drives reorganisation of the inactive SHR-chaperone heterocomplex [144,151,152], while simultaneously exposing the dimerisation interface, NLS peptide (cytoplasmic SHRs only) and DBD of the SHR [142,143]. Activated SHRs can then homo- (e.g. ERα-ERα) or hetero-dimerise (e.g. ERα-ERβ) [142,143,157–159], with cytoplasmic SHRs also undergoing nuclear translocation [142,143,151,159,160]. Intriguingly, the exact moment of dimerisation differs between SHRs, MR has been reported to dimerise after nuclear translocation [160] whereas GR appears to dimerise during translocation [159]. Regardless, once in the nucleus, SHR dimers dissociate from their chaperone heterocomplexes [151,152] and bind to HREs upstream of target genes, with each SHR monomer recognising one HRE half-site (Figure 3B) [46–48,62,64–68]. DNA-bound SHRs subsequently recruit transcriptional coregulators via the AF-1 and AF-2 regions in a tissue and cell-specific manner (Figure 4) [57,58,70–75,142,143].

SHRs can also undergo ligand-independent activation through phosphorylation, leading to receptor dimerisation, DNA binding and transcriptional coregulator recruitment (Figure 4). This is best characterised for ERα, where growth factor-related kinase cascades phosphorylate serine residues in the A/B domain (e.g. Ser^102^, Ser^104^, Ser^106^, Ser^118^, Ser^167^) [80–87,161–163]. Similar pathways have also been reported for AR [164–168] and PR [169–171], although these are less well defined. In contrast, ligand-independent transcriptional modulation has not been observed for GR or MR [87,172–178].

Beyond their direct genomic signalling roles, SHRs can function in a sequence-independent manner by associating with other DNA-bound transcription factors. This process, known as tethering, allows SHRs to regulate the expression of genes that lack HREs upstream of their promoter regions (Figure 4) [142,143]. Several examples of SHR tethering have been reported previously, including interactions between AP-1 (Fos/Jun) and GR [175], AP-1 and ERα [176–180], NF-κB and GR [181,182], and GR and MR [183]. However, more recent genomic data has begun to challenge aspects of these earlier studies, particularly regarding whether ER-GR cross-talk in luminal breast cancer is driven by direct GR tethering to ER-bound chromatin or alternative mechanisms. Specifically, Preković et al. [184] demonstrated GR was still capable of occupying previously identified ER co-bound regions even after degradation of the ER protein.

Several SHR-specific signalling mechanisms have also been reported. For instance, Hudson et al. [185] used X-ray crystallography to show that GR binds monomerically to negative GREs (nGREs) with the consensus sequence CTCC(N)_0-2_GGAGA. These nGREs inhibit GR dimer formation by positioning the D-box of each GR monomer on opposing DNA faces in antiparallel orientations [185], thereby enabling direct GR-mediated transrepression and inhibiting target gene expression (Figure 4). Despite this, work employing a constitutive GR monomer mutant (GR_mon_) reported severely impaired DNA binding [186], suggesting that GR function heavily depends on oligomerisation state [187]. Moreover, GR dimers have been reported to form tetramers upon DNA binding (Figure 4), supporting transcriptional activation and repression [187–189], and potentially facilitating GR access to distal regulatory elements via DNA looping [188,190].

### SHR coregulator recruitment and transcriptional control

Regardless of the genomic signalling mechanism, agonistic ligands promote the recruitment of transcriptional coactivators to the AF-2 hydrophobic groove of SHRs via NR box motifs [26,142,143], although interactions with the AF-1 region have also been documented [191]. Various SHR coactivators have been reported previously, including members of the steroid receptor coactivator (SRC) family, e.g. SRC1-3 [192–196], CBP/p300 [197] and MED1 [187,198,199]. Of these, SRC1-3, CBP and p300 induce target gene transcription in similar ways; either by converting facultative heterochromatin into transcriptionally active euchromatin via intrinsic histone acetyltransferase (HAT) activity [200–202], or by associating with general transcription factors (e.g. TFIIB) which in turn recruit RNA polymerase II to initiate target gene transcription [197,203–206]. Analogously, the MED1 subunit of the Mediator complex directly recruits RNA polymerase II [187,198,199]. SHRs have also been shown to interact with ATP-dependent chromatin remodelling complexes, such as SWI/SNF [207–211], which reposition or eject nucleosomes to form transcriptionally active euchromatin [212]. Moreover, various studies have reported interactions between SHRs and pioneer factors (e.g. FOXA1 [49,213–215], GATA3 [215,216], PBX1 [217], AP-1 [175–180]), which can bind to consensus DNA sequences within regions of facultative heterochromatin, and recruit chromatin remodelling complexes or HATs to drive chromatin opening and target gene expression [218].

In contrast, antagonistic SHs trigger transcriptional corepressors to be recruited to the SHR AF-2 hydrophobic groove via CoRNR box motifs [75]. Notably, some unliganded DNA-bound SHRs can also interact with corepressors, although these represent only a small subset of the total cellular SHR pool [219]. Several transcriptional corepressors have been described previously, including N-CoR [220] and SMRT [221]. These proteins typically suppress target gene expression by activating histone deacetylases (HDACs), which in turn drive chromatin compaction into transcriptionally silent heterochromatin [222]. Intriguingly, in contrast to prior work by Hudson et al*.* [185], other genomic studies have reported that transrepression of GR target genes occurs primarily without proximal GRE binding [223]. Instead, in these contexts, transcriptional repression by GR appears to involve indirect mechanisms, including the induction of epigenetic modifiers and long-range chromatin interactions, which likely contribute to the slower kinetics of GR transrepression relative to transactivation [224].

Crucially, SHR genomic signalling is highly context dependent, with chromatin accessibility, coregulator availability and transcription factor presence all shaping ligand responses. For instance, the selective estrogen receptor modulator tamoxifen silences ERα target gene expression in ERα+ breast cancer (BC) cells [225–230] but activates transcription in endometrium tissue via AF-1 [227–232]. Similarly, GR exhibits opposing roles in BC: acting as a tumour suppressor in luminal ERα+ BC [233–239], while promoting tumour formation in ERα- BC via epithelial-mesenchymal transition (EMT) induction, adhesion gene repression and apoptosis inhibition [233,240–243]. GR also blocks prostate cancer (PC) progression in anti-androgen sensitive cells [244,245], yet drives oncogenesis in castration-resistant PC cells [246–248]. This paradox highlights a critical gap in our understanding of the mechanisms by which cellular context shapes SHR signalling outcomes.

### Biomolecular condensates of SHRs

Following SH ligand stimulation, SHRs can concentrate into distinct foci within the nuclear compartment [249]. These SHR foci, later termed biomolecular condensates, were initially characterised in the 1980s by both transmission electron (TEM) and confocal microscopy [250–252]. More recently, the organisation of activated SHR nuclear complexes has been attributed to liquid-liquid phase separation, reviewed in detail in [249,252–255]. Discrete phase-separated SHR foci are predominantly thought to arise from multivalent interactions between the AF-1 and AF-2 regions, and SHR coregulators (e.g. weak interactions between AF-2 and the L-X-X-L-L NR box motif) [256–258]. One notable example of a coregulator that participates in SHR phase separation is MED1, which has been identified in ERα [259,260], GR [256] and AR [257,258] biomolecular condensates.

Currently, the role of SHR biomolecular condensates on transcriptional regulation is still the subject of ongoing research. For instance, Chen et al. [258] reported that expression of an AR-ΔIDR mutant (i.e. deletion of AR A/B domain) in LNCaP cells impaired AR condensate formation, inhibited AR chromatin binding and reduced the expression of *KLK2* and *NKX3-1* AR target genes. More recently, in a murine mammary adenocarcinoma cell line, 19% of GR condensates were found to colocalise with RNA polymerase 2 (Pol II) Ser5P, and 10% with Pol II Ser2P foci, implying a role in transcriptional initiation and elongation, respectively [261]. Thus, although recent advances have shed light on the role of SHR biomolecular condensates in transcriptional control, significant knowledge gaps remain, particularly regarding the exact mechanisms that drive this process. Clarifying these pathways may therefore provide novel strategies to target hormone-dependent pathologies [255].

### Non-genomic signalling mechanisms of SHRs

**Figure 5:**
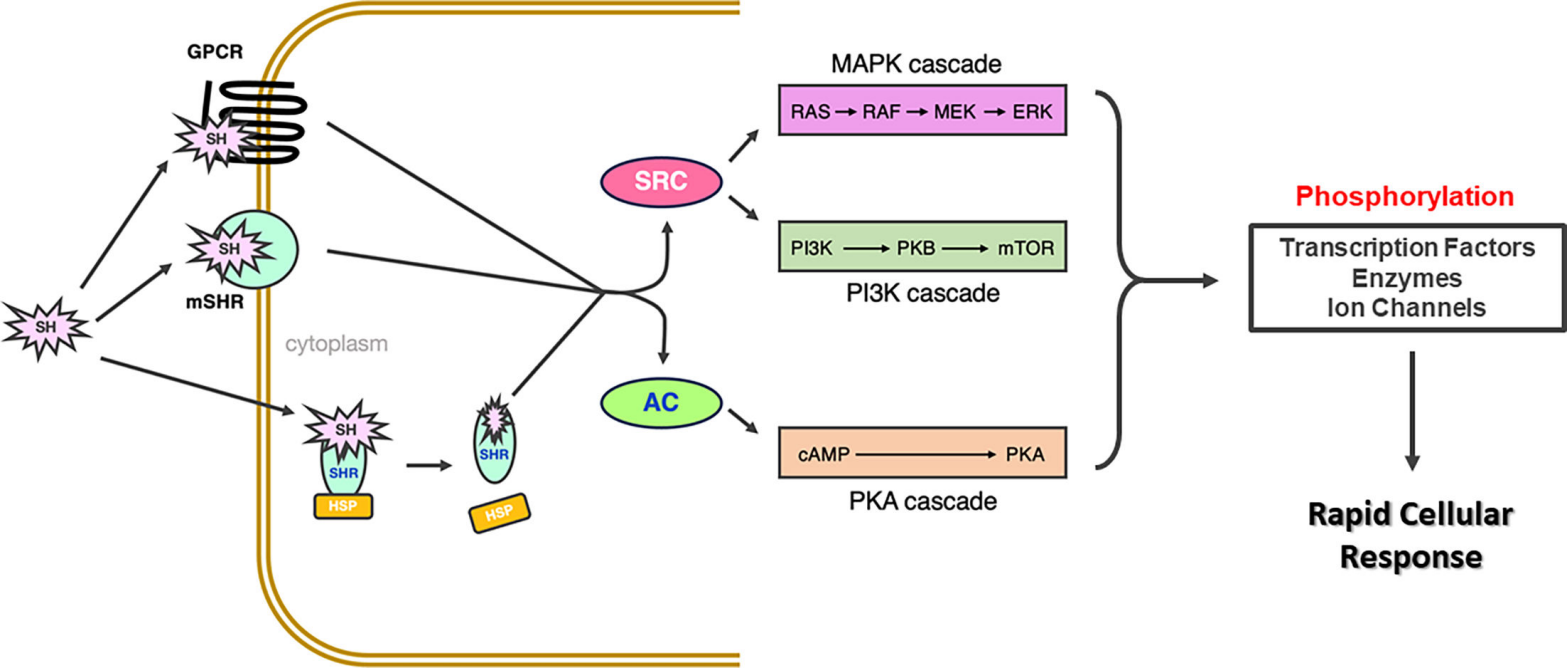
Schematic of non-genomic steroid hormone receptor (SHR) signalling mechanisms. Steroid hormones (SHs) can induce rapid cellular responses via cytoplasmic SHRs, membrane-bound SHRs (mSHR) and G-protein coupled receptors (GPCRs). These receptors activate adenylate cyclase (AC) and Src kinase, which subsequently induce the MAPK, PI3K and PKA kinase cascades. Downstream phosphorylation of target proteins (including transcription factors, enzymes and ion channels) modulates their activity and facilitates rapid cellular responses to SH stimulation. HSP, heat shock protein; MAPK, mitogen-activated protein kinase; PI3K, phosphoinositide 3-kinase; PKA, protein kinase A.

Non-genomic SHR signalling was first reported by Szego and Davis in 1967 [262], who observed a rise in uterine cAMP levels just 15 s after administering 17β-estradiol to ovariectomised mice. Since genomic SHR responses can take up to 60 min to manifest [263–266], this finding suggested a faster alternative signalling pathway existed. Subsequent work by Pietras and Szego [267] identified a membrane-localised ER (Figure 5), with similar receptors later documented for GR [24,268–270], MR [271,272], PR [273,274] and AR [275].

Alongside membrane-bound SHR variants, non-classical SH-activated receptors have been identified for AR [276], ER [277,278] and PR [279]. These are typically 7-transmembrane GPCRs that activate intracellular G-proteins and downstream signalling cascades upon SH binding (Figure 5). Moreover, cytosolic SHRs can also initiate intracellular signalling cascades independently to their genomic actions (Figure 5) [25].

Various intracellular signaling pathways are associated with rapid SH responses (Figure 5). For instance, membrane-bound PR (mbPR) [280,281], cytosolic PR [280], mbERα [282–284], GPCER [285–287] and mbGR [24,288–291] all activate Src, MAPK and PI3K cascades. Furthermore, SHRs can stimulate adenylate cyclase, leading to cAMP production and PKA activation, as shown for mbER [292], mbPR [293] and GPCGR [294]. All of these cascades phosphorylate key target proteins (e.g. transcription factors, enzymes, ion channels), altering their activity to yield rapid cellular responses [25].

SHRs may also act as RNA-binding proteins, rapidly modulating mRNA stability in response to SH stimulation. This effect has been reported for ERα [295] and GR [296], although it lies beyond the scope of this review.

## Importance of studying SHR interactions with proteins and DNA on chromatin

Given the fundamental role that DNA and protein interactions hold in regulating genomic SHR signalling, advances in the methods used to study these interactions have closely paralleled, and enabled, our understanding of SHR biology.

Much of our early understanding of SHR-protein and SHR-DNA interactions came from low-throughput biochemical assays, although classical high-throughput methods including Yeast Two Hybrid (Y2H) systems also played an important role. More recently, the emergence of high-throughput genomic and proteomic platforms has transformed the field, enabling more systems-based investigations into SHR chromatin-bound complexes.

The application of these high-throughput techniques to study changes in SHR-DNA binding and SHR-protein interactions has enabled insights into a range of topics, including how SHR interactions are reprogrammed during tumorigenesis [297] and how this links to patient outcomes [298]. Beyond cancer, these approaches have been instrumental in elucidating SHR signalling pathways [223], and their roles in neuronal development [299], metabolic disorders and cardiovascular diseases [300]. This review will discuss how SHR-protein and SHR-DNA interactions can be studied both *in vitro* and *in vivo*.

## Methods for studying interactions between proteins and SHRs

### Historical techniques for studying SHR-protein interactions

#### Yeast Two Hybrid (Y2H) systems

**Figure 6:**
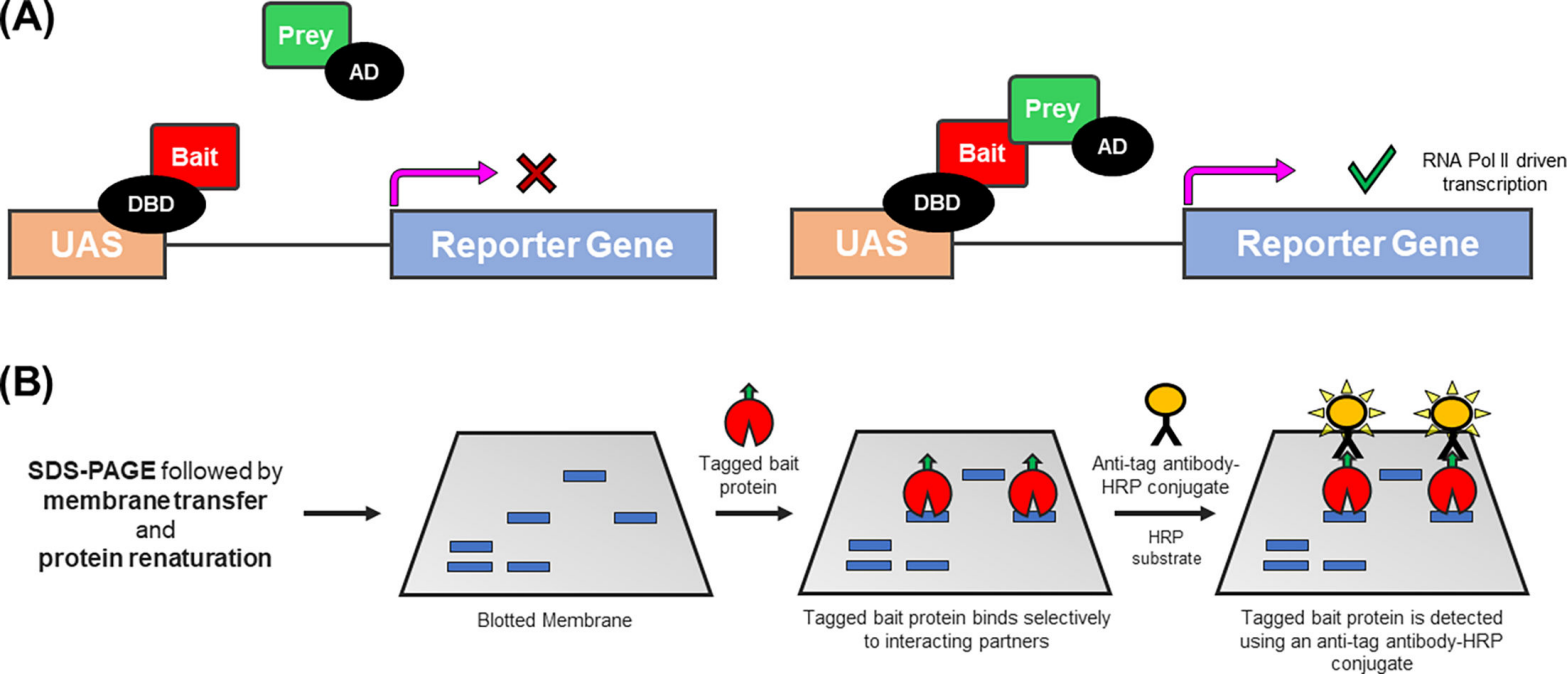
schematic overview of classical techniques for detecting protein-protein interactions. **(A**) Yeast two-hybrid (Y2H) assays. The bait protein is fused to the DNA-binding domain (DBD) of a transcription factor (e.g. GAL4-DBD), while the prey protein is fused to a transcriptional activation domain (AD), such as GAL4-AD or HSV-VP16. When co-expressed in yeast cells, any interaction between the bait and prey proteins brings the DBD and AD into close proximity, driving the recruitment of RNA polymerase II to the upstream activator sequence (UAS) of a reporter gene (e.g. *LacZ*). Subsequent expression of a reporter protein (e.g. b-galactosidase) can be detected using chromogenic substrates like X-Gal. (**B**) Far western blotting (FWB). Proteins are separated by SDS-PAGE, transferred to a membrane, and renatured. The membrane is then incubated with a tagged bait protein, which functionally replaces the primary antibody used in conventional WB. Binding partners (i.e. prey) of the bait protein are subsequently detected using an anti-tag antibody-HRP conjugate, enabling visualisation of specific bait-prey interactions.

Yeast Two Hybrid (Y2H) assays detect *in vivo* protein-protein interactions by reconstituting a functional transcription factor in yeast cells [301]. Most Y2H systems employ a *LacZ* reporter gene under the control of an upstream activator sequence (UAS). Two fusion constructs are required for this: the bait, comprising gene X fused to the DNA-binding domain (DBD) of the GAL4 transcription factor; and the prey, in which gene Y is fused to a transactivation domain (AD), such as GAL4-AD or HSV-VP16. Interaction between the bait and prey brings the DBD and AD into proximity, facilitating RNA polymerase II (Pol II) recruitment to the *LacZ* UAS, and driving β-galactosidase expression (Figure 6A) which can be quantified using chromogenic substrates (e.g. X-Gal).

Y2H assays are popular for their simplicity, low cost and ability to capture interactions within living cells, providing greater physiological relevance than many *in vitro* methods. Moreover, large-scale screening experiments are possible using cDNA-derived prey libraries [301–303]. However, non-specific binding events can yield false positive results, while protein misfolding or absent post-translational modifications (e.g. *N*-glycosylation) can lead to false negatives [301,304]. Furthermore, fusion proteins such as bait and prey can adopt aberrant structures or subcellular localisations, potentially altering the interactome observed by Y2H [305,306].

Various studies have utilised Y2H systems to explore SHR-protein interactions. For instance, Hong et al. [193] employed a GAL4-based Y2H *LacZ* assay to show that GRIP1 interacts with the LBDs of mGR, hER and hAR, while related studies identified LBD associations with SRC1-3 [307] and SMRT [308]. Using the same approach, Wang et al. [309] demonstrated estrogen-induced hER dimerisation, whereas Ballaré et al. [310] reported a direct interaction between hERα and hPR-B.

#### Far western blotting

Far western blotting (FWB) has played a largely historical role in investigating SHR-protein interactions [311]. In this method, proteins are separated by denaturing SDS polyacrylamide gel electrophoresis (PAGE), transferred to a membrane and renatured via stepwise removal of denaturing agents. The membrane is then blocked and probed with a tagged protein of interest (POI), with interactions detected using an antibody against the POI’s tag (Figure 6B).

FWB is especially useful for identifying novel protein-protein interactions, as it bypasses the need for POI-specific primary antibodies and can be combined with proteomic approaches such as liquid chromatography tandem mass spectrometry (LC-MS/MS) [312,313]. However, because renaturation is often incomplete [311], protein interactions that depend on native 3D structures might be missed. Additionally, non-specific binding events can cause false positive results, while the need to overexpress the tagged POI adds time and complexity. Regardless, FWB has contributed to the historical characterisation of SHR interactomes [314–316].

### Current biochemical methods for investigating SHR-protein interactions

#### Proximity ligation assays

**Figure 7:**
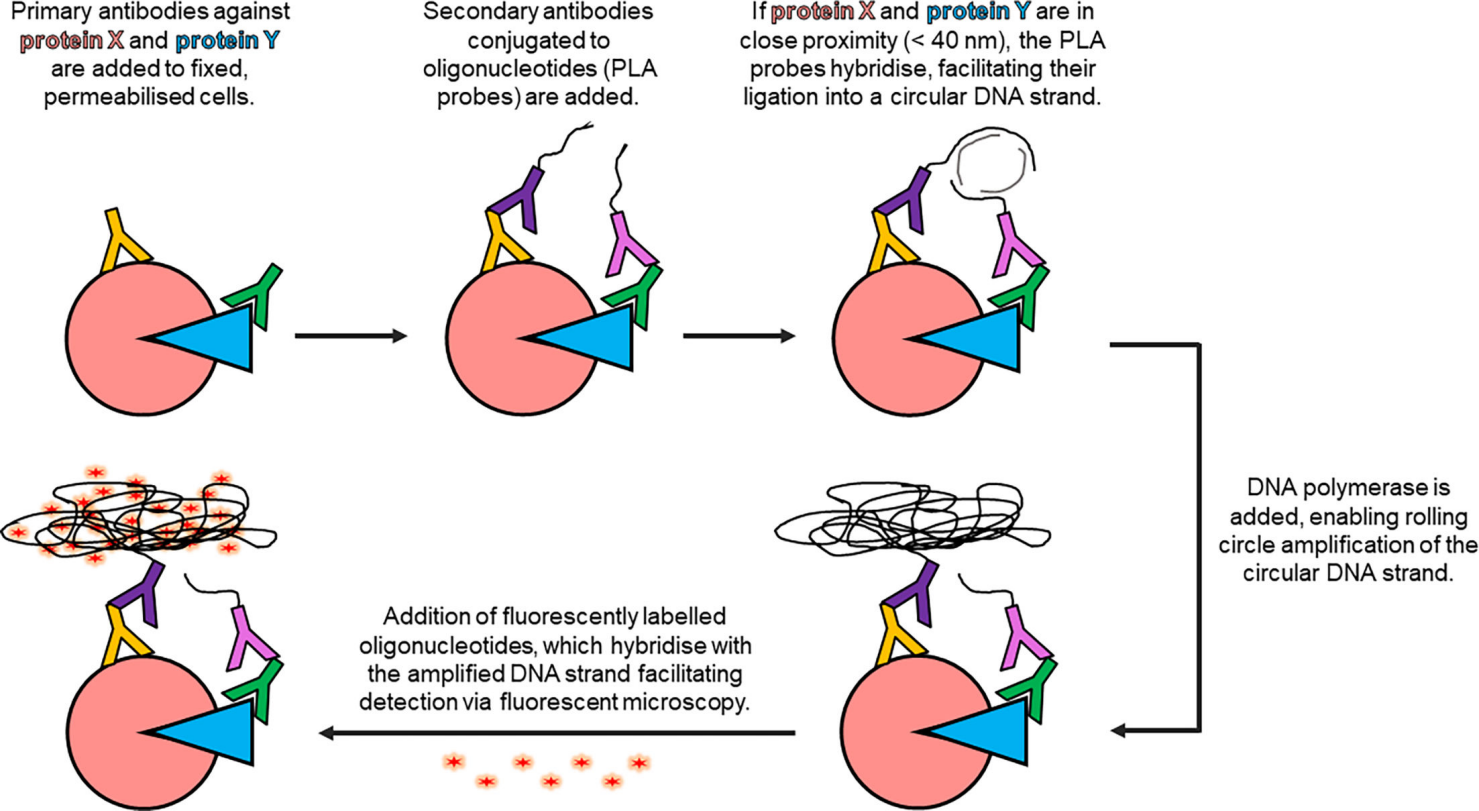
Schematic representation of the proximity ligation assay (PLA) workflow.

Proximity ligation assays (PLA) enable the *in situ* detection of protein-protein interactions [317]. After fixation and permeabilisation, cells are incubated with primary antibodies raised against the target POIs (hereafter ProteinX and ProteinY), followed by secondary antibodies tagged with complementary oligonucleotides (termed PLA probes). If ProteinX and ProteinY are in close proximity (<40 nm), the PLA probes can hybridise, enabling their ligation into a circular DNA strand which is amplified via rolling circle replication. Fluorescently labelled probes subsequently bind to the amplified DNA molecule, producing a distinct fluorescent dot that represents a single binding event (Figure 7).

Several notable studies have employed PLA to characterise SHRs. For instance, work from our lab [318] used PLA to show that hERα and the ZMIZ1 transcriptional coactivator occupy the same transcriptional complex in ERα+ breast cancer cells. PLA has also been used to examine the subcellular localisation of ER homo-/hetero-dimers in response to agonistic and antagonistic ligand stimulation [319], alongside characterising the ERα/PI3K/Src complex [320] and validating qPLEX-RIME findings [321].

While PLA can be applied to proteins *in situ*, there are limitations. High levels of background signal present the need for several robust positive and negative controls. Moreover, PLA requires multiple antibodies to be validated and optimised.

#### Pulldown assays

Pulldown assays, also known as affinity purifications (AP), are a well-established technique for identifying protein-protein interactions. While several types of pulldown assay have been described (Table 1:), they all generally follow a common principle. In brief, a tagged protein of interest – termed the bait – is produced and immobilised on a solid support, typically beads or a column, through its tag. A sample containing potential interacting partners – termed the prey – is then introduced to the solid phase, either in a purified form or as part of a crude cell lysate. Following a series of washes to remove non-specifically bound proteins, bait-prey complexes are eluted from the solid phase for analysis, for example via SDS-PAGE, western blotting, or proteomics-based approaches.

**Table 1:** Examples of biochemical pulldown assays.

Pulldown assay	Technical overview	Advantages	Disadvantages
Glutathione S transferase (GST) Pulldown [322–324]	The bait construct is generated by fusing the protein of interest (X) to glutathione S-transferase (GST). Following overexpression and purification from *Escherichia coli*, the bait protein is immobilised on a glutathione-coated solid-phase support and incubated with the prey. When required, bait-prey complexes are eluted from the solid-phase support using a buffer containing glutathione (GSH).	Elution of bait-prey complexes from solid-phase support is straightforward. GST fusion proteins are typically soluble, stable and resistant to aggregation. This means small fragments of protein X can be expressed with ease, enabling domain-mapping experiments.	The GST tag is large (~26 kDa), which may alter the structure or function of protein X. GST fusion proteins are usually isolated from *Escherichia coli* cells, which do not fully replicate eukaryotic protein folding or post-translational modifications. As such, some bait-prey interactions might be missed.
Biotin/Streptavidin Pulldown [325–333]	The protein of interest (X) is chemically or enzymatically tagged with biotin to generate the bait protein. Notably, biotinylation can be performed *in vivo*, or following purification, depending on the study. Bait proteins are then immobilised on streptavidin-coated solid-phase supports, before incubating with the prey. Bait-prey complexes are then eluted from the solid support through linker cleavage, protease digestion, or streptavidin denaturation.	Biotin tags are small (~244 Da), so do not affect the structure/function of protein X. Since biotinylation can occur *in vivo* or after isolation, protein X can be expressed in various systems, including mammalian cells. The biotin-streptavidin binding affinity is incredibly high (*Kd* ~ 10^-14^ M), meaning very little bait protein is displaced during the wash steps. As such, this technique is commonly used to study transient or low-affinity bait-prey interactions.	The elution of biotinylated bait-prey complexes from streptavidin is difficult without enzymatically cleavable linkers. Protease digestion can yield unwanted streptavidin peptides. Denaturing conditions destroy higher order protein structures, including those in the bait-prey complex.
His_6_, FLAG and HA Pulldowns [334–338]	A small genetically encoded peptide tag (e.g. His_6_, FLAG, HA) is fused to the protein of interest (X) to yield the bait protein. After overexpression and purification, the bait is immobilised to a solid-phase support coated in Ni^2+^-NTA (His_6_), anti-FLAG antibodies (FLAG), or anti-HA antibodies (HA). Following prey incubation, bait-prey complexes can be eluted from the solid-phase support using buffers containing imidazole (His_6_), FLAG peptides, or HA peptides. Low pH buffers can also be used (FLAG and HA).	Peptide tags are small ( < 1.1 kDa), so are not likely to alter the structure or function of protein X. Peptide tags are compatible with various expression systems, including mammalian cells.	The affinity of His_6_ for Ni^2+^-NTA coated solid-phase supports is relatively low (*Kd* ~ 10^-6^ M), which might cause bait-prey leaching during washing. The affinity of anti-FLAG and anti-HA antibodies for their respective tags can vary between products and batches.

Pulldown assays are an elegant and cost-effective approach for detecting protein-protein interactions. The *in vitro* nature of these assays allows for buffer optimisation (e.g. pH, salt or ligand concentration), which can maximise bait-prey complex recovery for downstream analysis. However, the overexpression and purification of tagged bait proteins is not trivial [339]. Moreover, false positives may result from non-specific protein-protein binding events, while false negatives can arise due to improper bait protein folding, the absence of specific interacting partners (i.e. multi-protein complexes), or missing post-translational modifications.

Despite these limitations, pulldown assays are widely used in SHR research. For example, Liao et al. [308] utilised a GST pulldown domain-mapping assay to show that hAR interacts with the ID-2 domain, but not the ID-1 domain, of the corepressor SMRT. Ballaré et al. [310] employed a similar GST pulldown experiment to show an interaction between hERα and the hPR-B ERID-1/ERID-2 domains, which in turn activates Src kinase. In contrast, Ishmael et al. [296] utilised a biotin-streptavidin pulldown assay to illustrate the RNA-binding properties of hGR. In this study, *CCL2* and *CCL7* mRNA transcripts were biotinylated, incubated with whole cell lysates and treated with the glucocorticoid budesonide. Biotin-streptavidin AP and agarose gel electrophoresis revealed a significant reduction in transcript abundance after budesonide treatment, consistent with hGR-mediated mRNA decay.

#### Immunoprecipitation (IP) and co-immunoprecipitation (CoIP)

**Figure 8:**
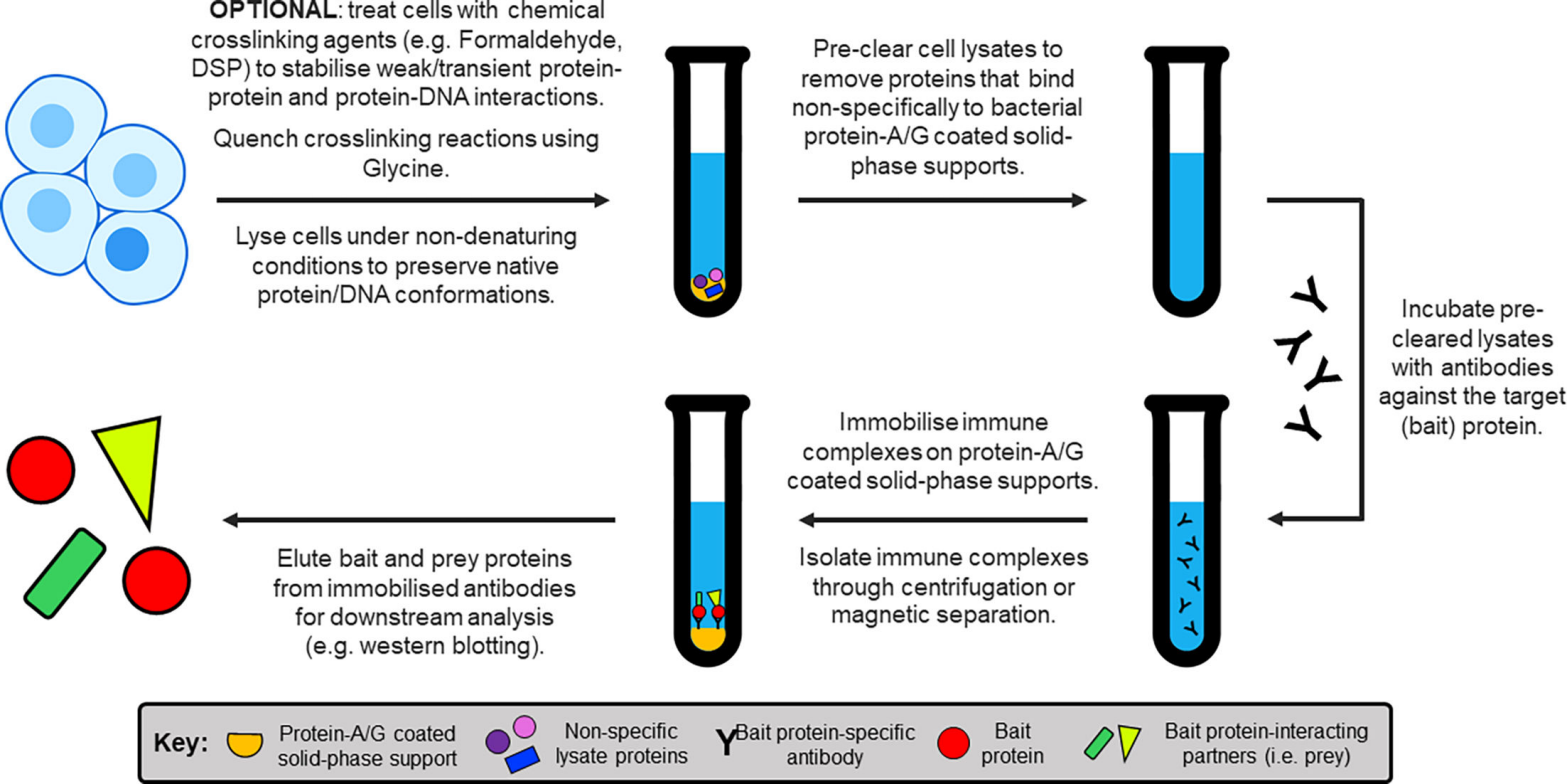
Schematic representation of the workflow for immunoprecipitation (IP) and co-immunoprecipitation (CoIP) assays.

Immunoprecipitation (IP) and co-immunoprecipitation (CoIP) are the gold standard for SHR-protein interaction studies. While these techniques are methodologically similar, they have distinct purposes: IP isolates a single target protein for analysis, whereas CoIP aims to purify the interacting partners (i.e. the prey) of a specific target protein (i.e. the bait). In both IP and CoIP, cell lysates are initially prepared under non-denaturing conditions to preserve native protein structures and protein-protein interactions. An optional pre-clearing step can then be performed to remove proteins that bind non-specifically to the solid-phase support. Antibodies raised against the bait protein are subsequently added to the cell lysate, enabling the formation of immune complexes (e.g. IgG-bait or IgG-bait-prey). These complexes are immobilised on solid-phase supports coated with bacterial protein A [340] or protein G [341], isolated from whole cell lysates through centrifugation or magnetic separation, and eluted for downstream analysis (Figure 8). For lower-affinity protein-protein or protein-DNA interactions, chemical cross-linking reagents (e.g. formaldehyde, DSP) can be applied before lysis to stabilise weak or transient biomolecular complexes.

The main advantage of IP and CoIP over other classical techniques is their ability to isolate endogenous protein complexes without the use of tags or fusion proteins. However, both IP and CoIP rely on high-affinity anti-bait antibodies which are not always available. Moreover, without additional experiments, IP-based studies cannot easily distinguish direct from indirect protein-protein interactions.

IP and CoIP are nearly ubiquitous across SHR-protein interaction studies. For instance, Veldscholte et al. [342] used CoIP coupled with western blotting to demonstrate rapid HSP dissociation from hAR in prostate cancer cells following androgen treatment. Similar studies have provided evidence for the interaction of hERα and hGR in breast cancer cells dependent on glucocorticoid and estrogen stimulation [343], alongside associations between hGR and hPR [344], and hERα and hMR [345]. As such, IP/CoIP-based assays are often used to validate key findings from high-throughput proteomics approaches [215,321].

### Current biophysical approaches for investigating SHR-protein interactions

Biophysical techniques provide a powerful method for quantifying protein-protein interaction parameters, offering detailed insights into the binding affinity (*K_d_*), interaction distance and stoichiometry of binding events. These approaches enable real-time monitoring of molecular interactions, both *in vitro* and *in vivo*. Among the wide range of biophysical tools available for studying SHR-protein interactions, the most commonly used are Fӧrster resonance energy transfer (FRET), surface plasmon resonance (SPR), isothermal titration calorimetry (ITC) and various fluorescence spectroscopy methodologies, as summarised in Table 2:.

**Table 2:** Examples of biophysical methods for studying biomolecular interactions.

Biophysical tool	Technical overview	Advantages	Disadvantages
Fӧrster resonance energy transfer (FRET) [346,347]	The first protein of interest (i.e. the bait) is tagged with a donor fluorophore, while the second protein of interest (i.e. the prey) is tagged with an acceptor fluorophore. If these fluorophores are in close proximity (typically < 10 nm), and their excitation and emission spectra overlap, the donor can transfer some excitation energy to the acceptor via non-radiative dipole-dipole coupling. This results in the excitation of the acceptor fluorophore and the emission of detectable fluorescence.	Especially useful for studying changes in protein dynamics *in vitro and in vivo*. The distance between donor and acceptor fluorophores (i.e. the interaction distance) can be quantified using FRET efficiency, which follows an inversely proportional relationship.	Requires the bait and prey proteins to be labelled with fluorophores, which can be technically challenging and may alter their structure and/or function. Fluorophores must be carefully selected to ensure spectral overlap and prevent fluorescence bleed-through. Only works with short-range interactions ( < 10 nm). Photobleaching can lead to a reduction in FRET efficiency over time.
Surface Plasmon resonance (SPR) [346,348]	The bait protein is immobilised on a gold-plated sensor chip, with a laser being directed at the rear surface under conditions of total internal reflection. Surface plasmons (electrons) in the chip absorb energy from the incident laser light, producing a characteristic dip in reflected light intensity (the absorbance band). When potential interacting partners (prey) are flowed over the chip, any binding events alter the refractive index, in turn deflecting the angle of the absorbance band. These angle changes can then be measured to quantify molecular interactions.	Highly sensitive technique capable of detecting binding events using minimal amounts of prey protein. Enables real-time, label-free monitoring of the kinetics of molecular interactions.	Equipment and consumables required are expensive. Immobilisation of the native, interaction- competent bait protein on the sensor-chip can be challenging. Regenerating the sensor-chip for future use is not trivial.
Isothermal titration calorimetry (ITC) [349–351]	ITC provides direct thermodynamic insights into protein-protein interactions by quantifying the heat exchanged during binding events. The instrument consists of two chambers: a reference cell filled with buffer, and a sample cell containing the bait protein in the same buffer. The prey protein is subsequently injected into the sample cell in small increments, while the temperature changes associated with bait-prey interactions are monitored. From these measurements, key thermodynamic parameters can be derived, such as the enthalpy change (**Δ**H), dissociation constant (*K_d_*) and binding stoichiometry.	Capable of highly accurate thermodynamic measurements, including binding affinity. Enables real-time, label-free monitoring of binding event kinetics. The bait and prey proteins are in solution rather than being immobilised, so they are more likely to be in their native state.	Protein-protein interactions must bring about measurable temperature changes, rendering ITC unsuitable for very strong or very weak binding events. ITC requires large quantities of highly purified bait and prey proteins.
Fluorescence correlation spectroscopy (FCS) [352,353]	FCS is a powerful microscopy tool used to study protein dynamics. In brief, a tightly focused laser beam, usually generated by confocal or two-photon microscopy, is directed towards a small diffraction-limited spot within cells (~200–300 nm in diameter). As fluorescently labelled molecules (e.g. tagged SHRs) move into and out of this foci, the fluorescence intensity fluctuates up and down. Temporal fluctuations in fluorescence can then be analysed to unveil key molecular behaviours; for instance, the diffusion speed, or if the molecule of interest interacts with other partners (as indicated by prolonged dwell times within the foci).	Provides a quantitative way to measure molecular diffusion rates, concentration and binding kinetics. High temporal resolution, allowing rapid molecular dynamics to be captured (μsec – msec timescale). Effective at low fluorophore concentrations, minimising perturbation to the system under study.	Requires labelling with bright, photostable fluorophores to prevent photobleaching and minimise background noise. Can miss key heterogeneous molecular dynamics outside of the diffraction-limited foci.
Fluorescence Cross-Correlation spectroscopy (FCCS) [352,353]	FCCS is an extension of FCS that utilises two or more spectrally distinct fluorophores to determine whether different molecules interact. As in FCS, temporal fluctuations in intensity are measured for each fluorescently tagged species. The fluctuation traces from each fluorophore are then compared to reveal co-ordinated molecular behaviour (e.g. simultaneous diffusion, prolonged co-dwell times) indicative of molecular interactions.	Directly measures interactions between two or more differently labelled molecular species. Can quantify the interaction strength (*Kd*) between fluorescently labelled molecules *in vivo*. Can detect co-diffusion and co-binding events with a very high sensitivity. Retains all the advantages of FCS.	Requires careful optimisation of fluorophores to prevent spectral overlap. More complex microscopy setup and more challenging data analysis than FCS. Retains all the disadvantages of FCS.
Number and brightness (N&B) [352,354]	N&B is an extension of FCS in which a region of interest (ROI) is repeatedly raster scanned with a confocal microscope. Frame-to-frame fluctuations in the fluorescence intensity arise due to changes in the number of fluorescent molecules within the ROI (i.e. due to diffusion), and from differences in their oligomeric state. As multimers exhibit more fluorescence and larger intensity fluctuations than monomers, the molecule’s oligomeric state can be determined by analysing the variance and mean fluorescence intensity over time.	High spatial resolution, allowing molecular oligomerisation states to be determined and localised within living cells. Relatively straightforward data analysis. Effective at low fluorophore concentrations, minimising perturbation to the system under study	Molecule(s) of interest must be labelled with bright, photostable fluorophores to reduce photobleaching and photoblinking. Requires a high-quality, low-noise confocal microscope to minimise background fluorescence.
Single molecule tracking (SMT) [355]	SMT is an advanced super-resolution microscopy tool that enables individual fluorescently labelled molecules to be imaged and tracked over time. When analysed, these data provide insights into molecular movement (e.g. free diffusion, confined, directed motion), alongside the duration of binding events. As such, SMT facilitates the measurement of heterogeneous diffusion behaviour and binding dynamics at the single-molecule level.	Direct, real-time visualisation of individual fluorescently labelled molecules. Detects heterogeneity in molecular dynamics (e.g. confined *vs*. directed motion, fast *vs*. slow diffusion). Generates spatially resolved trajectory track data, revealing where key molecular behaviours occur at a subcellular level.	Requires molecule(s) of interest to be labelled with bright, photostable fluorophores to reduce photobleaching and photo-blinking. Molecular tracking accuracy is limited by signal-to-noise ratio, and motion blur. Challenging data analysis that often needs specialised tracking algorithms.

Various groups have used *in vitro* biophysical tools to explore SHR-protein interactions. For example, Neo et al. [356] utilised SPR to demonstrate an interaction between hERα and the transcription factor Sp1. In this study, biotinylated DNA fragments containing either intact or scrambled EREs and Sp1-binding sites were immobilised on gold-plated sensor chips. Subsequent SPR analysis revealed that hERα could localise to DNA harbouring a scrambled ERE through an interaction with Sp1. In a separate study, Copik et al. [357] used ITC to characterise the interaction between hGR and the TBP coactivator, showing that hGR binds TBP with a high nanomolar affinity, and in a 2:1 stoichiometric ratio (consistent with dimeric hGR engaging monomeric TBP). Parent, Gunther and Katzenellenbogen utilised FRET to monitor interactions between the LBDs of hERα [358] or hAR [359] and members of the SRC transcriptional coactivator family, facilitating the design of small molecule inhibitors.

Fluorescence spectroscopy approaches have also generated insights into SHR behaviours within living cells. For instance, Mikuni et al. [360] and Stasevich et al. [361] utilised fluorescence correlation spectroscopy (FCS) to monitor intranuclear GR dynamics, revealing fast-diffusing receptor populations, and slower-diffusing species indicative of chromatin binding. Building on this, Stortz et al. [362] used FCS to demonstrate GR exhibits both fast (chromatin lag time < 60 ms) and slow (chromatin lag time > 200 ms) nuclear diffusion, with agonistic SHs driving longer chromatin residence times. These results imply GR (and SHRs more broadly) may undergo rapid DNA binding, sliding, or searching prior to stable chromatin engagement. Complementing these FCS findings, single-particle tracking (analogous to SMT) of GR condensates by the same group [256] showed that while most GR foci were chromatin-confined, two additional subsets were detected: one heavily constrained in close proximity to the nucleoli, and another located more distally that explored far larger nuclear regions. Additionally, Savatier et al. [363] used FCCS to estimate the *in vivo K_d_* of ER and the TIF2 transcriptional coactivator under different ligand conditions (agonist < 6 nM; antagonist > 3 μM; unliganded ~ 200 nM).

### Current proteomics approaches for investigating SHR-protein interactions

#### Proximity labelling mass spectrometry

Proximity labelling mass spectrometry (PL-MS) is a powerful method for identifying novel protein-protein interactions. In brief, PL-MS uses a bait-enzyme fusion protein to covalently label interacting partners (i.e. prey) with distinct molecular tags, enabling their enrichment via affinity purification and identification by mass spectrometry. Typically, liquid-chromatography tandem mass spectrometry (LC-MS/MS) is employed. Affinity-purified prey proteins are enzymatically digested (e.g. with trypsin), generating peptides that are separated by LC and analysed using electrospray-ionisation (ESI) tandem MS. The resulting peptide mass spectra are then matched to proteomics databases to identify the original prey proteins. For reviews of MS-based proteomics tools, see Han et al. [364], Yates et al. [365] and Dau et al. [366].

Of the various PL-MS techniques available, BioID and APEX have emerged as the most widely adopted (Table 3:). While their application to SHRs remains limited, Lempiäinen et al. [373] used BioID to identify both known (e.g. MED1, SRC1-3) and novel (e.g. BCOR, TLE3) interacting partners of hGR and hAR. Similarly, Agbo et al. [374] used BioID to characterise the interactome of hERα. To our knowledge, APEX has not yet featured in SHR research, though it has been applied successfully to study other proteins, such as the angiotensin II type 1 G-protein coupled receptor (AT1R) [375].

**Table 3:** Overview of proximity labelling mass spectrometry (PL-MS) techniques for studying protein-protein interactions.

PL-MS method	Technical overview	Advantages	Disadvantages
Proximity-Dependent Biotin Identification (BioID) [367–370]	The bait protein is fused to a mutant *E. coli*-derived promiscuous biotin ligase (BirA^R118D^; BirA*), which facilitates the biotinylation of prey proteins within a 10 nm radius of the bait-BirA* fusion protein. Biotinylated prey is subsequently isolated via biotin-streptavidin affinity purification, before subjecting to tryptic-digest LC-MS/MS (or similar proteomic MS tools) for identification.	Can be performed *in vitro* and *in vivo*. Affinity purification of biotinylated prey proteins is relatively straightforward. Captures all bait-prey binding events within a 10 nm radius of the bait-BirA* fusion protein, including those that are weak or transient.	The bait-BirA* fusion protein and the native bait protein may exhibit different expression levels or subcellular localisations, potentially affecting the interactome. The BirA* biotin ligase is slow, requiring long incubations (18–24 hours) with biotin to sufficiently label prey proteins. This limitation has been addressed with new optimised BirA mutants that can tag prey proteins with biotin in under 10 minutes – for example, TurboID and MiniTurbo [369]. All BirA enzymes selectively attach biotin to primary amines, meaning only prey proteins with accessible lysine ε-amino groups or N-termini will be tagged.
Proximity-Dependent Ascorbate Peroxidase (APEX) [371,372]	The bait protein is fused to an engineered ascorbate peroxidase enzyme (APEX), which uses H_2_O_2_ and biotin-phenols to generate highly reactive biotin-phenoxyl radicals. These radicals biotinylate electron-rich amino acid residues (e.g. Tyr, Trp, His, Cys) within a 20 nm radius of the bait-APEX fusion protein. Biotinylated prey proteins are subsequently isolated via biotin-streptavidin pulldown and identified by tryptic-digest LC-MS/MS (or another comparable proteomic MS approach). Notably, a catalytically enhanced APEX mutant (APEX^A134P^; APEX2) is now commonly used.	Affinity purification of biotinylated prey proteins is relatively straightforward. Captures all bait-prey binding events within a 20 nm radius of the bait-APEX fusion protein, including those that are weak or transient. APEX enzymes sufficiently label prey proteins with biotin in under 1 minute, rendering APEX suitable for temporal proteomics experiments.	The bait-APEX fusion protein and the native bait protein may exhibit different expression levels or subcellular localisations, potentially affecting the interactome. All APEX enzymes selectively attach biotin to electron-rich amino acid residues, limiting the number of prey proteins that can be labelled. All APEX enzymes require cytotoxic H_2_O_2_ as a cofactor, making *in vivo* studies challenging.

#### Rapid immunoprecipitation mass spectrometry of endogenous proteins

**Figure 9:**
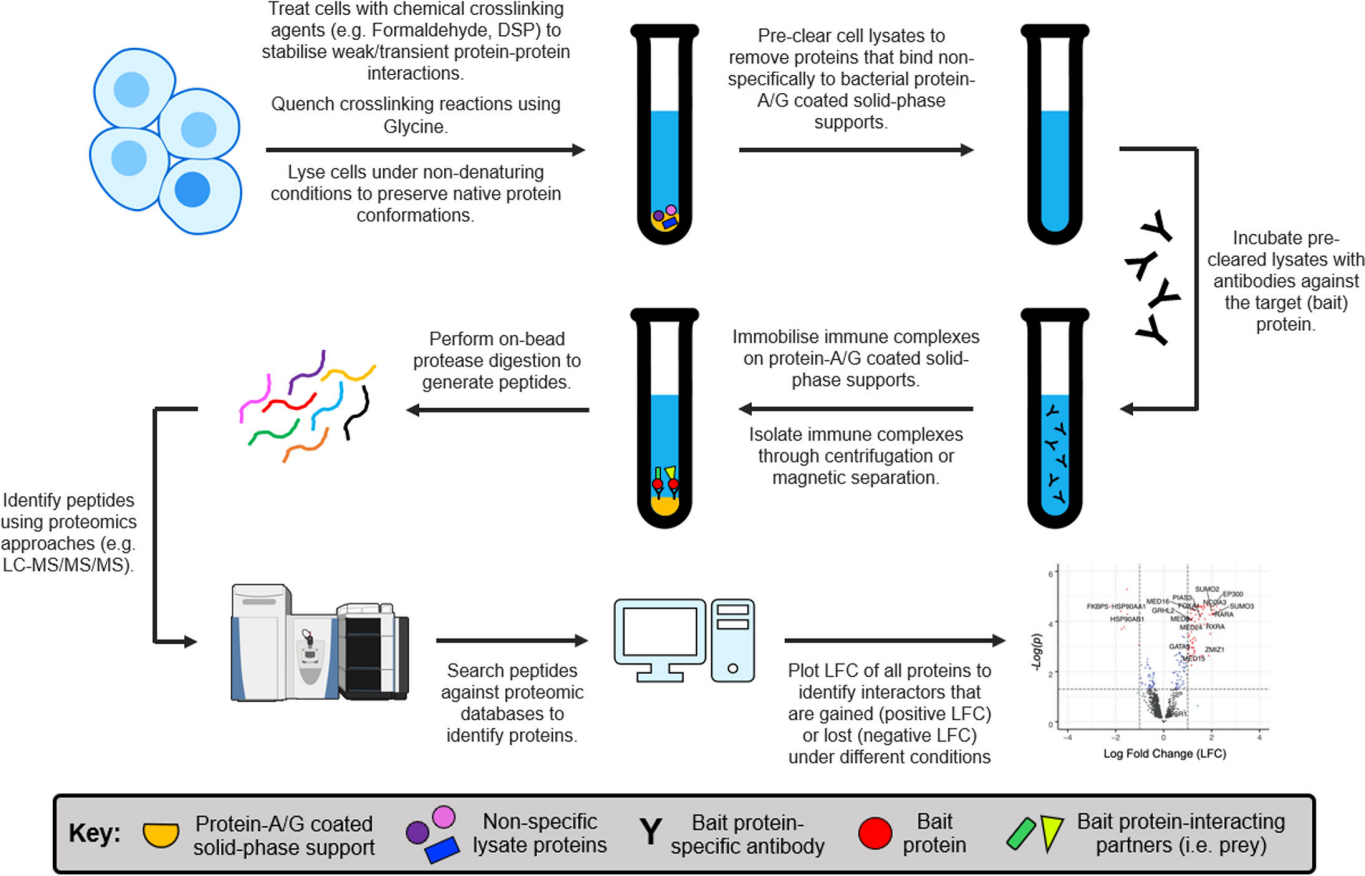
Schematic of the rapid immunoprecipitation mass-spectrometry of endogenous proteins (RIME) workflow. Notably, the lysis step can be modified to extract cellular nuclei, allowing interactions between SHRs and nuclear proteins to be investigated at the chromatin. In such cases, sonication is performed to shear the chromatin prior to immunoprecipitating the protein of interest. LFC plot was adapted from [318]. LFC, log-fold change.

Rapid immunoprecipitation mass spectrometry of endogenous proteins (RIME) integrates cross-linking immunoprecipitation (XL-IP) with mass spectrometry to identify novel interacting partners of specific bait proteins [215,321,376,377]. In this method, live cells are treated with chemical cross-linkers (e.g. DSP, formaldehyde) to preserve native protein-protein and protein-DNA complexes, before lysing under non-denaturing conditions. The stabilised bait-prey assemblies are then isolated via IP and enzymatically digested, typically on-bead using trypsin, to generate unique peptides from each protein. Alternative proteases, including chymotrypsin, elastase, proteinase K, AspN and GluC, can also be used to expand peptide coverage [366]. These peptides are subsequently analysed through LC-MS/MS, or another comparable proteomic MS approach [364–366], with the resulting spectra searched against proteomic peptide databases to identify interacting partners of the bait protein (Figure 9). This core RIME workflow can also be coupled with various peptide labelling methods to enable the simultaneous analysis of multiple different samples, as outlined in Table 4:.

**Table 4:** Overview of rapid immunoprecipitation mass spectrometry (RIME) techniques for studying protein-protein interactions.

RIME method	Technical overview	Advantages	Disadvantages
Rapid Immunoprecipitation Mass *S*pectrometry of Endogenous Proteins**(RIME)** [215]	Cells are typically crosslinked with formaldehyde to preserve protein–protein and protein–DNA interactions, followed by chromatin shearing. An antibody targeting the endogenous bait protein is used to immunoprecipitate associated protein complexes directly from nuclear or chromatin-enriched lysates. After extensive washing, bound proteins are digested on-bead and identified by LC–MS. RIME enables the unbiased identification of proteins associated with a native bait under near-physiological conditions, without the need for tagging or overexpression.	Enables identification of interacting partners of endogenous proteins in their native cellular context. Avoids artefacts associated with protein overexpression or tagging. Established ChIP-seq reagents translate to RIME.	Largely qualitative interaction data. IgG-based background subtraction can be over-stringent without quantification, excluding true interactors detected in IgG controls.
Stable Isotopic Labelling by Amino Acids in Culture **(SILAC-RIME)** [215,377]	Cells are cultured in media lacking specific amino acid residues (e.g. arg, Lys) and are supplemented with isotopically distinct *light* or *heavy* variants (i.e. ^12^C/^13^C, ^14^N/^15^N) that differ in mass. Separate cell populations – such as those exposed to different drug treatments – incorporate these labelled amino acids during protein synthesis, resulting in condition-specific isotopic labelling of proteins. These isobaric labels allow peptide abundances to be quantitatively compared between conditions via RIME, enabling changes in prey protein levels across samples to be measured.	Enables identification novel interacting partners of endogenous bait proteins over IgG control. Can provide quantitative data about how protein-protein interactions change under different conditions.	SILAC-RIME is restricted to a maximum of 3 conditions, as only three types of isotopic media – *light*, *medium* and *heavy* – are available for cell culture. Isotopically defined *heavy* media is more expensive than standard culture media.
Quantitative Multiplexed **(qPLEX-RIME)** [321]	Separate cell populations (e.g. those treated with different drugs) are processed using the standard RIME protocol. Following on-bead enzymatic digestion, peptides from each sample are labelled with isobaric tandem mass tags (TMTs), typically at the N-terminus or lysine ε-amino groups. These TMTs are identical in mass so signal is additive at the MS1 level. However, upon fragmentation, TMTs release unique reporter ions that enable the differentiation of peptides originating from distinct samples during MS2 or MS3. As such, qPLEX-RIME allows several conditions to be examined within a single MS run, with the relative intensities of the TMT reporter ions facilitating quantitative comparisons of peptide – and corresponding prey protein – abundance between samples.	Expanded capabilities of quantitative RIME beyond three concurrent samples. Multiplexing reduces sample acquisition time. The capacity for simultaneous condition analysis is restricted only by the number of isobaric TMTs available, with up to 34-plex currently supported.	TMT labelling costs increase with higher multiplexing of samples. Pooled reference channels are required for quantitative comparision across multiple TMT-plexes. MS2-based TMT quantification is prone to ratio compression, partially mitigated by MS3 approaches.
Label-free DIA-NN enabled RIME **(DIANNeR)** [378].	Separate biological conditions are processed independently using a modified RIME workflow, without isotopic or chemical labelling. Following on-bead digestion, peptides are analysed by data-independent acquisition (DIA) on a Bruker timsTOF HT mass spectrometer. Peptide identification and quantification are performed using DIA-NN, which enables a spectral library-free approaches to achieve high sensitivity and reproducibility. Relative protein abundances are inferred from peptide intensities across samples, enabling quantitative comparison of RIME interactomes in a label-free manner.	Does not require isotopic or chemical labelling, reducing cost and experimental complexity. Scalable to large numbers of conditions and biological replicates, with no technical limit on sample number. Compatible with limited quantity samples where metabolic labelling is not feasible.	Quantification is sensitive to batch effects and requires careful experimental design. Pooled reference samples are required to control for run-to-run variation when analyses are not performed concurrently.

RIME-based methodologies have been widely used to map SHR interactomes. For instance, seminal work by Mohammed et al. [215] employed SILAC-RIME in MCF-7 cells to identify hERα-binding partners following treatment with either 17β-estradiol or tamoxifen, confirming several known hERα interacting partners (e.g. SRC3, FOXA1 and GREB1). Later work by the same group applied qPLEX-RIME to expand the MCF-7 hERα interactome [321], with various novel binding partners being detected following 17β-estradiol stimulation (e.g. CBX3, NIPBL, FOXK1). This work also revealed tamoxifen treatment reduced hERα binding to some proteins (e.g. SRC3, GREB1), while enhancing interactions with others (e.g. SMRT, HDAC2, several SWI/SNF subunits). SILAC-RIME has also been used to map the interactomes of hPR-A and hPR-B in T47D-C42 cells with or without progestin, identifying several previously reported cofactors common to both hPR isoforms (e.g. FKBP5, PARP1), alongside novel isoform- and ligand-state-specific interacting partners [379]. More recently, a label-free DIA-NN enabled RIME (DIANNeR) analysis of hGR across a diverse range of cell types has been reported, including normal untransformed cells and patient-derived xenografts (PDXs), providing a more detailed understanding of the complex hGR interactome [378].

### Future methods for studying protein-protein interactions

#### UV-driven cross-linking coupled with RIME (UVXL-RIME)

**Figure 10:**
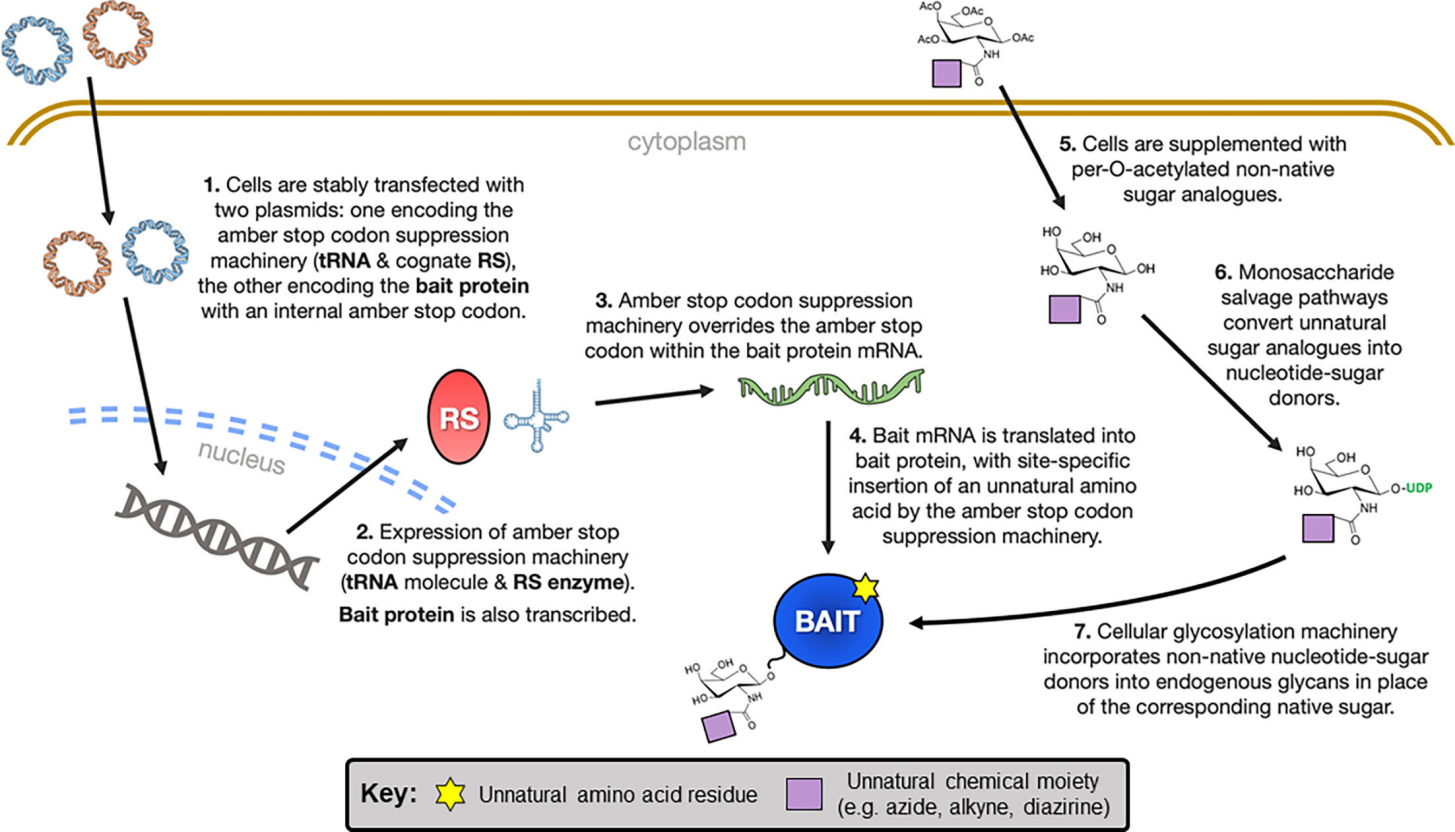
Schematic representation of amber stop codon suppression (steps 1–4) and metabolic glycan labelling (steps 5–7). For site-specific insertion of unnatural amino acids to be possible, the amber stop codon suppression machinery must be engineered to recognise the amino acid of interest. Furthermore, non-native sugar analogues are initially per-O-acetylated to enhance their lipid solubility, but are rapidly deacetylated in the cytoplasm. RS, aminoacyl tRNA synthetase.

A key limitation of current RIME-based tools is their tendency to isolate large multi-protein complexes, making it difficult to distinguish direct interactions from indirect interactions, and limiting structural insights into bait-prey binding events. These challenges could feasibly be addressed by tagging the bait protein with short-range UV-inducible photocross-linking probes that would functionally replace the chemical cross-linking step in the RIME workflow. Although several approaches can be used to achieve this, two promising strategies are metabolic glycan labelling (for *N*/O-glycosylated proteins) [380–386] and amber stop codon suppression [387–395] (Figure 10).

Metabolic glycan labelling involves supplementing cells with monosaccharide analogues that contain unnatural chemical groups (e.g. azides, alkynes). These non-native sugars are taken up by the cells, processed into nucleotide-sugar donors by monosaccharide salvage pathways [384] and inserted into endogenous glycan chains in place of the native form of the sugar by the cellular glycosylation machinery [380–386]. In contrast, amber stop codon suppression employs a genetically engineered tRNA/aminoacyl tRNA synthetase (RS) pair to override an internal amber stop codon (UAG) in the bait mRNA transcript, facilitating the site-specific incorporation of unnatural amino acids into the bait protein [387–395]. If these unnatural amino acids or sugars carry UV-inducible photocross-linking groups (e.g. diazirines [396]), they could covalently capture bait-interacting partners within a small radius ( < 2 nm) of their insertion site. Theoretically, such an approach would remove large multi-protein complexes from the RIME workflow while providing structural data about bait-prey binding events.

Notably, both amber stop codon suppression [395] and metabolic glycan labelling [384] have recently been implemented within mammalian cells, demonstrating their compatibility with complex eukaryotic systems. As such, coupling these methods with existing RIME workflows represents a feasible and promising next step.

#### Subcellular protein-localisation coupled with RIME (LOPIT-RIME)

Current RIME methods aim to enrich for bait-prey interactions that occur at the chromatin. Since many SHRs have both non-genomic and RNA-binding roles, technologies capable of systematically mapping SHR interactomes across multiple subcellular compartments would be of great interest. One possibility is the integration of RIME with the LOPIT protocol outlined by Dunkley et al. [397,398]. In LOPIT, cell lysates are fractionated via detergent-based chromatin enrichment and density gradient ultracentrifugation. Proteins from each fraction are then enzymatically digested to generate peptides, which are labelled with TMTs (as in qPLEX-RIME; see Table 4:) and analysed using LC-MS/MS/MS, enabling the accurate identification of proteins within sub-organellar structures. In principle, combining RIME and LOPIT to create a spatial RIME approach builds on the strengths of both methodologies, since the existing qPLEX-RIME workflow could easily be modified with LOPIT prior to peptide generation. Such a technique would facilitate research into how SHR interactomes differ between subcellular compartments (e.g. nucleus *vs*. cytosol).

## Methods for studying interactions between DNA and SHRs

### Electrophoretic mobility shift assays

Electrophoretic mobility shift assays (EMSAs), or gel shift assays, are a classical *in vitro* technique used to characterise SHR-DNA interactions [399]. This method requires only small quantities of the protein of interest (POI) and DNA [400], although prior knowledge of the POI’s DNA binding sequence is needed. In brief, the POI and DNA are mixed, loaded into agarose or polyacrylamide gels [401] and separated by electrophoresis. Due to their larger size, POI-DNA complexes migrate more slowly through the gel than free linear DNA [402], allowing certain binding properties (e.g. dissociation constant; *K_d_*) to be estimated. Crucially, because POI-DNA interactions are conformation dependent, both must remain in their native state [401,403].

EMSAs have played a pivotal role in defining SHR binding to HREs. For instance, Kuntz and Shapiro [404] used the technique to assess how dimerisation influences hERα DBD affinity for consensus and non-consensus ERE half sites. Interestingly, dimeric DBD fragments had a higher affinity for both consensus and non-consensus EREs, although weak monomeric binding (*Kd* ~ 160 nM) was observed due to the ‘caging effect’, in which the gel maintains a high local concentration of POI and DNA that stabilises low-affinity interactions [405–407].

Bourdeau et al. [408] examined how ERE nucleotide substitutions altered hERα and mERα binding. While both receptors still bound consensus EREs harbouring single nucleotide polymorphisms, further substitutions markedly reduced binding. However, because this study used whole-cell extracts rather than purified ER, a ‘super-shift’ assay, where POI-specific antibodies are used to further slow complex migration, is needed to confirm DNA binding specificity [409].

EMSAs were also employed by Nguyen et al. [410] to ascertain how the hAR P-box G577R mutation, linked to partial androgen insensitivity, alters GRE binding. Intriguingly, the mutant receptor displayed reduced or no affinity for several consensus and non-consensus GREs.

#### Chromatin immunoprecipitation (ChIP)

**Figure 11:**
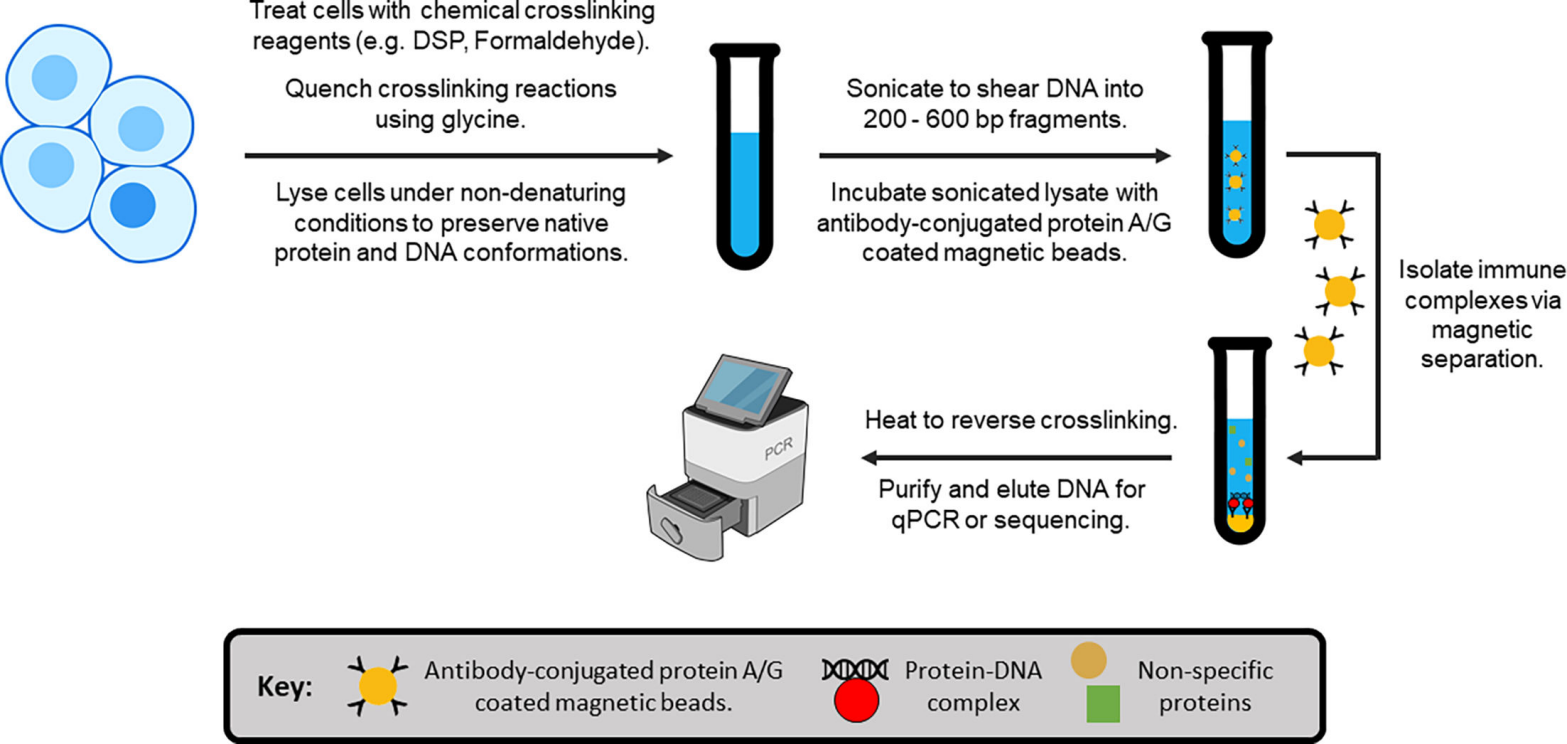
Schematic overview of chromatin immunoprecipitation (ChIP). Cells are chemically cross-linked to preserve native protein-DNA complexes prior to isolating the chromatin, and shearing via sonication. Target protein-DNA complexes are then immunoprecipitated using antibody-conjugated Protein A/G magnetic beads. The chemical cross-links are subsequently reversed through heating, and the purified DNA is analysed via qPCR or high-throughput sequencing approaches.

Chromatin immunoprecipitation (ChIP) is a fundamental technique for detecting protein-DNA interactions *in vivo* [411], typically requiring millions to tens-of-millions of input cells [412]. In brief, ChIP enriches genomic fragments that have specific transcription factors or histones bound to them. This method is similar to IP and RIME, except cross-linked chromatin is fragmented into short fragments prior to bait protein purification [413–416]. The chemical cross-linking is then reversed through heating, releasing the DNA for purification. Once isolated, the DNA is analysed by sequencing (ChIP-seq) or quantitative polymerase chain reaction (ChIP-qPCR) (Figure 11) [49,417]. As such, ChIP produces a temporal ‘snapshot’ of where specific proteins are bound to the chromatin. Although not discussed here, ChIP can also be integrated with other methods, such as chromatin conformation capture (e.g. 3C), reviewed by Fullwood et al. [418] and Han et al. [419], to investigate SHR-induced conformational changes in the 3D structure of genomic DNA [420–423].

ChIP-seq is especially useful for identifying the genomic origin of isolated DNA fragments, although it is also the costliest ChIP assay [424]. In ChIP-seq, purified DNA fragments are sequenced using next-generation sequencing (NGS) technology, as reviewed by Satam et al. [425]. The sequenced fragments are then mapped to a reference genome, enabling the genomic origin of the fragments to be identified [426]. Bioinformatic analysis is then used to identify ‘peaks’ – regions of the genome where mapped sequences are overrepresented in the ChIP sample relative to background controls – thus indicating where the transcription factor of interest is bound (termed ‘peak calling’) [427,428]. However, ChIP-seq normalisation remains challenging, as differences in sequencing depth, IP efficiency, and global changes in transcription factor occupancy between conditions can confound quantitative comparisons. [429,430]

ChIP-seq has been widely used to investigate SHR-genome interactions in disease states such as cancer. For instance, Wilson et al. [431] utilised ChIP-seq to identify the androgen response element (ARE) in prostate cancer cell lines. Additionally, Welboren et al. [432] used ChIP-seq to map the genomic binding of ERα and RNA Polymerase II (Pol II) in MCF-7 cells treated with 17β-estradiol (E2), or the antagonistic ligands tamoxifen and fulvestrant (a selective ER degrader) [433,434]. For E2-activated genes, both antagonists reduced ERα and Pol II binding. In contrast, for E2-repressed genes, tamoxifen induced their down-regulation, while fulvestrant increased Pol II binding, highlighting the power of ChIP-seq to detect cistrome changes under different conditions.

Beyond cell lines, ChIP-seq has also been successfully applied to patient-derived material. For example, Ross-Innes et al. [298] deployed ChIP-seq on primary breast tumours to identify changes in hERα-chromatin binding that were associated with different patient outcomes. Similarly, Severson et al. [435] mapped ERα-, AR-, PR- and GR-chromatin binding in breast tumour samples from male and female patients to elucidate sex-mediated cross-talk between SHRs, revealing these SHRs to be strongly colocalised at genomic loci in both genders.

ChIP-qPCR continues to play a role in low-throughput genomic studies and for validating ChIP-seq data [436–438]. For example, Bourdeau et al. [408] used ChIP-PCR to verify novel ERE sites within the genome. In place of the NGS approaches used in ChIP-seq, ChIP-qPCR quantifies qPCR fluorescence to measure transcription factor binding at a single locus [428]. Although ChIP-qPCR has a lower cost to entry than ChIP-seq, it requires prior knowledge of the transcription factor’s DNA binding sequence to design sequence-specific primers.

Importantly, the quality of ChIP data heavily depends on the quality of the antibody [439]. ChIP-validated polyclonal and monoclonal antibodies do exist for ER, AR, MR and GR [440–443], although not all antibodies are validated for the cell types used. Additionally, batch-to-batch variations in polyclonal antibodies can affect specificity, while monoclonal antibodies carry a higher risk of epitope masking. As a result, some studies use monoclonal antibody pools, although this increases cost. Poor antibody quality can also result in off-target capture, which can mask on-target capture. Therefore, validating SHR-specific antibodies in relevant cell lines is a critical step in ChIP research [444].

#### CUT&RUN and CUT&TAG

As described above, ChIP experiments are ‘high input’, and thus cannot detect SHR binding in smaller cell populations. While low-input ChIP-seq methods do exist (Table 5:), to our knowledge their use in SHR research is limited. Two commonly used low-input alternatives are Cleavage Under Targets and Release Using Nuclease (CUT&RUN) [448] and Cleavage Under Targets and Tagmentation (CUT&TAG) [449], which require hundreds of cells, or potentially single cells, respectively [450].

**Table 5:** Overview of low input ChIP methodologies for studying protein-DNA interactions.

Low input ChIP method	Technical overview	Advantages	Disadvantages
Quick and quantitative ChIP **Q^2^-ChIP** [445]	Modified ChIP assay. Key differences in the protocol include: Cross-linking cells in suspension (adherent cells would require trypsinisation). Includes a ‘tube shift’ step to ensure background material that has adhered to the tube wall is not recovered. Cross-link reversal, proteinase K digestion of proteins and DNA purification are combined into a single step.	Requires ≥ 100,000 cells. Rapid (completed in 1 day). Higher efficiency than standard ChIP.	Reducing the amount of input chromatin increases the amount of background noise (although background subtraction is possible). Only one IP can be performed per DNA sample. Uses formaldehyde for protein-DNA cross-linking, which is carcinogenic and toxic.
MicroChIP **µChIP** [446]	Similar to Q^2^-ChIP, but modified as follows: For cross-linking, biopsies are thawed in formaldehyde and sodium butyrate. Tissue aggregates are removed.	Low input – can be performed on as few as 100 cells. Applicable to tissue biopsies (both fresh and frozen). Rapid (completed in 1 day). Higher efficiency than standard ChIP. Cheaper than ChIP-seq.	
Ultra-low-input micrococcal nuclease-based native ChIP **ULI-NChIP** [447]	Modified ChIP assay. Key differences in the protocol include: Use of a micrococcal nuclease to fragment the DNA. Use of low-cycle PCR amplification to minimise over-representation of some fragments.	Requires ≥ 100,000 cells. Can achieve similar resolution to samples of more than 1,000,000 cells. Minimises sequencing costs. Does not require formaldehyde cross-linking.	Reducing the amount of input chromatin increases the amount of background noise.

**Figure 12:**
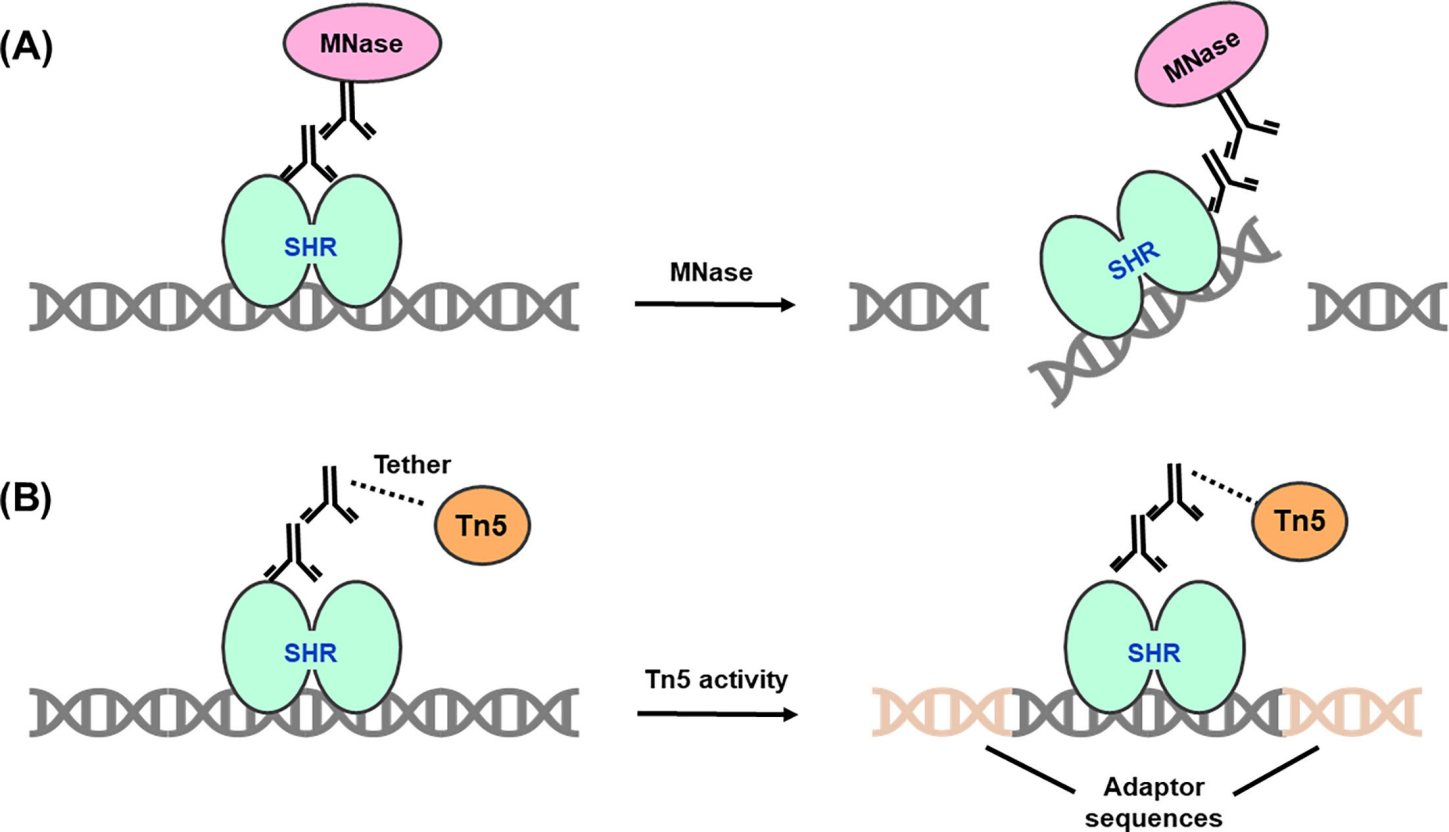
Overview of CUT&RUN and CUT&TAG methodologies. **(A**) In CUT&RUN, primary antibodies raised against the protein of interest (SHR) are added to fixed, permeabilised cells. Secondary antibodies conjugated to micrococcal nuclease (MNase) are then added. MNase cuts the genomic DNA either side of the SHR-binding site, releasing DNA fragments that can be identified through NGS. (**B**) CUT&TAG is similar to CUT&RUN, except the secondary antibody tethers a Tn5 transposase to the protein of interest (SHR) binding site. Tn5 integrates adaptor sequences either side of the SHR binding site, enabling amplification via PCR and identification through NGS.

In CUT&RUN (Figure 12A), permeabilised cells are incubated with primary antibodies against the protein of interest (POI), followed by secondary antibodies fused to micrococcal nuclease enzymes (MNase) [448]. Upon POI-DNA binding, MNase cleaves the chromatin flanking the POI-binding site, releasing DNA fragments for identification through NGS. Gegenhuber et al. [451] used this method to identify estrogen-responsive genes in the brains of mice, revealing several ERα binding sites that promote sex differences.

CUT&TAG is similar to CUT&RUN, except the secondary antibody tethers a transposase (Tn5) to POI-DNA binding sites (Figure 12B) [452–454]. Tn5 integrates adaptor sequences into the genome that flank the POI-DNA binding site, enabling amplification by qPCR or detection via NGS technologies. CUT&TAG has been used by Guo et al. [455] to verify co-recruitment of SET with hERα to EREs.

Advantageously, CUT&RUN and CUT&TAG do not use formaldehyde cross-linking, which is commonly used in conventional ChIP-seq and can introduce false positive POI–DNA interactions [455,456], thereby reducing background noise compared with ChIP-seq. However, CUT&RUN and CUT&TAG are more susceptible to nonspecific peaks due to differences in peak calling [457]. Moreover, both techniques suffer from the same limitations as ChIP with regards to antibody requirements.

#### Single-molecule tracking (SMT)

Because ChIP, CUT&RUN and CUT&TAG only capture a temporal ‘snapshot’ of SHR-DNA interactions, transient TF-chromatin binding events can be missed [458]. Real-time imaging tools such as single-molecule tracking (SMT) [355], described in Table 2:, provide a feasible way to address this limitation.

Accordingly, SMT is increasingly being used to study heterogeneous TF binding behaviours. In the context of SHRs, Paakinaho et al*.* [459] employed HaloTag-receptor fusion proteins alongside SMT to explore transient interactions of GR, AR, PR and ER with the chromatin [460]. In the absence of SHs, only a small proportion (~15%) of SHRs were chromatin-bound at any one time. Following SH stimulation, this proportion increased substantially (~50%), although under both ± SH conditions, most receptor binding events exhibited rapid exchange dynamics (chromatin residence times of ≤ 1 second).

#### Future methods for studying protein-DNA interactions

Several techniques have been proposed as alternatives to ChIP, CUT&RUN and CUT&TAG. One such method, Calling Cards, goes beyond current single-cell TF binding tools (e.g. single-cell CUT&TAG [453]) by simultaneously generating binding and transcriptomic data at the single-cell resolution [461–464], thereby allowing highly heterogeneous TF behaviours to be studied.

**Figure 13:**
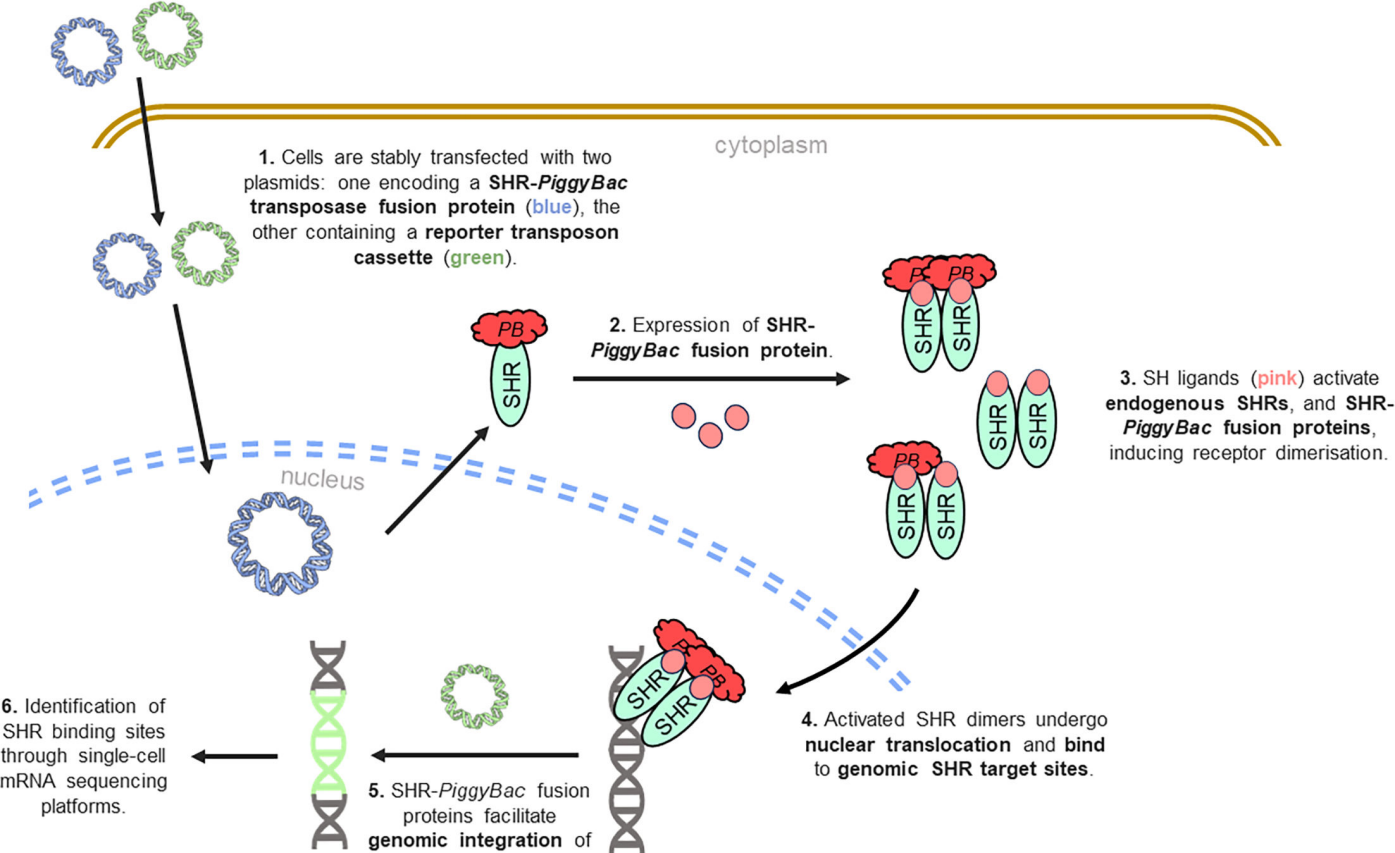
Schematic of the potential application of a Calling Cards approach for investigating SHR binding patterns. Self-reporting transposon (SRT) cassettes are stably integrated into the genome at SHR target sites by SHR-PBase fusion proteins. Each SRT cassette contains a promoter and reporter gene flanked by PiggyBac inverted terminal repeat (ITR) sequences, but lacks a polyadenylation sequence (PAS). Consequently, when the reporter gene is transcribed from the SRT promoter, RNA polymerase II continues into the adjacent genomic DNA until it encounters a cryptic PAS, thereby incorporating the SRT into the mRNA of SHR target genes. These mRNAs transcripts can then be analysed as using droplet-based single-cell sequencing platforms. PBase, PiggyBac transposase.

*In situ* Calling Card approaches employ TF-hyperactive PiggyBac transposase fusion proteins, which integrate a Self-Reporting Transposon (SRT) cassette at genomic TF binding sites (Figure 13). The SRT contains a promoter and reporter gene flanked by terminal repeats, but crucially lacks a polyadenylation sequence (PAS). Consequently, when the reporter gene is expressed, RNA polymerase II continues into the adjacent genomic region until it reaches a cryptic PAS, thereby incorporating the SRT into the mRNA of TF-target genes [461,462]. Transfected cells can then be harvested via standard bulk or single-cell RNA-seq protocols [462,463] to reveal TF binding information comparable with ChIP-seq, while also generating transcriptomic data when single-cell methods are used [464].

## Conclusions

The study of protein-protein and protein-DNA interactions remains central to advancing our understanding of SHR biology in both normal and cancerous tissues.

Methods for exploring protein-DNA interactions (Supplementary Figure s1) are continually evolving, allowing the study of increasingly smaller cell populations, with single-cell studies beginning to emerge. These advances will provide critical insights into the role SHR signalling plays at a cellular level, whether between different cell types within a single tissue (e.g. mammary gland), or among cells within heterogeneous tumours [465].

The development of methods for studying SHR-protein interactions at low cell numbers has been more challenging, primarily due to the lack of signal amplification techniques (e.g. PCR for DNA-based studies), and our reliance on shotgun MS-based proteomics. Nonetheless, advances in alternative protein detection technologies [466], and the ongoing development of large-scale quantitative protein cross-linking methods [467] show great promise.

In summary, years of technological progress has provided us with a widely successful toolkit for exploring the role of SHRs in gene expression. Although the methods we have discussed typically have higher requirements for cellular material than RNA-/DNA-seq methods, we are optimistic that ongoing innovations will not only overcome these challenges but also deepen our understanding of SHR function and regulation across various fields.

## Supplementary material

online supplementary figure 1.

## Open Access

Open access for this article was enabled by the participation of University of York in an all-inclusive Read & Publish agreement with Portland Press and the Biochemical Society.

## Abbreviations

AF-1 / AF-2: activation function 1 / 2 (domains)
AR: androgen receptor
BC: breast cancer
DBD: DNA-binding domain
DIANNeR: Label-free DIA-NN enabled RIME
EMT: epithelial–mesenchymal transition
ER: estrogen receptor
ERE: chromatin immunoprecipitation
GCs: glucocorticoids
GR: glucocorticoid receptor
HAT: histone acetyltransferase
HDAC: histone deacetylase
IDR: intrinsically disordered region
LBD: ligand-binding domain
MCs: mineralocorticoids
MR: mineralocorticoid receptor
PC: prostate cancer
PL-MS: proximity labelling mass spectrometry
PR: progesterone receptor
Pol II: RNA polymerase II (Pol II)
RIME: rapid immunoprecipitation mass spectrometry of endogenous proteins
RTK: Receptor tyrosine kinase
SHRs: steroid hormone receptors
SHs: steroid hormones
SMT: single-molecule tracking
qPLEX-RIME: quantitative multiplexed RIME
