# Supplementary material for: ChIP happens: from biochemical origins to the modern omics toolbox for understanding steroid hormone receptors
Source: Biochem J. 2026 Feb 2;483(2):247–88. doi: 10.1042/BCJ20253216 (PMC12905503; doi:10.1042/BCJ20253216)
Supplement: online supplementary figure 1. [file bcj-483-2-BCJ20253216-s001.docx]

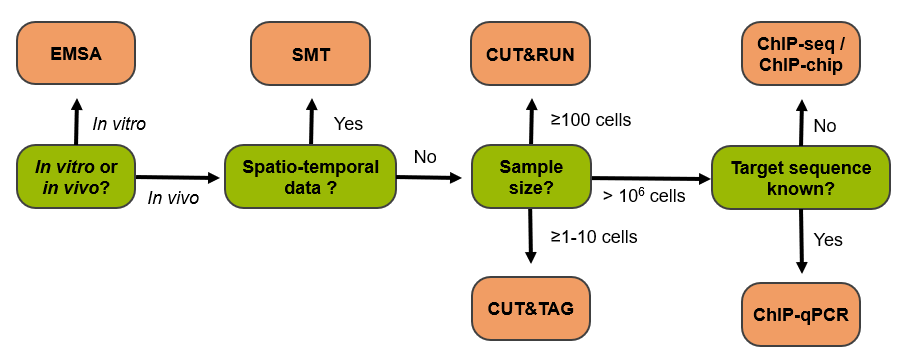


**Supplementary Figure 1 – Flowchart depicting the main techniques for examining protein-DNA interactions.**
